# Supplementary material for: Pharmacologic ATF6 activating compounds are metabolically activated to selectively modify endoplasmic reticulum proteins
Source: eLife. 2018 Aug 7;7:e37168. doi: 10.7554/eLife.37168 (PMC6080950; doi:10.7554/eLife.37168)

Supplementary Materials and Methods

Lentivirus Production

Vesicular stomatitis virus glycoprotein (VSV-G) pseudotyped lentiviral particles were produced by co-transfecting 293T cells with the structural plasmids necessary for virus production (Rev, RRE, and VSVG) along with three separate pLKO.1 shRNA lentivirus constructs for the gene (Sigma). Lenti-X 293T cells were transfected using calcium phosphate for 16 hr, after which the media was removed and replaced with fresh media. Media containing viral particles was collected at 48 hr post-transfection. Virus producing cells were removed by centrifugation at 1500 rpm for 5 minutes at ambient temperature. The supernatant was filtered through a 45 µm filter to remove cells. The media containing virus was used immediately for transfection or stored at -80 °C until use. The shRNA sequences are in Table S1.

Table S1: Sequences of shRNA

Non-silencing	
PDIA1 (P4HB)	1. CCGGGTGTGGTCACTGCAAACAGTTCTCGAGAACTGTTTGCAGTGACCACACTTTTTG<br>2. CCGGAGGTGAAATCAAGACTCACATCTCGAGATGTGAGTCTTGATTTCACCTTTTTTG,<br>3. CCGGTGCTGTTCTTGCCCAAGAGTGCTCGAGCACTCTTGGGCAAGAACAGCATTTTTG
PDIA3	1. CCGGGCTTACTATGATGTGGACTATCTCGAGATAGTCCACATCATAGTAAGCTTTTTTG<br>2. CCGGCGATTTGCACATACGAATGTTCTCGAGAACATTCGTATGTGCAAATCGTTTTTTG<br>3. CCGGCCAACACTAACACCTGTAATACTCGAGTATTACAGGTGTTAGTGTTGGTTTTTTG
PDIA4	1. CCGGCCTGAGAGAAGATTACAAATTCTCGAGAATTTGTAATCTTCTCTCAGGTTTTTG<br>2. CCGGCTTGGTCCTAAATGATGCAAACTCGAGTTTGCATCATTTAGGACCAAGTTTTTG<br>3. CCGGGCTTGTGTTGACCAAAGAGAACTCGAGTTCTCTTTGGTCAACACAAGCTTTTTG
PDIA5	1. CCGGGCTCCTGAAGAAGGAAGAGAACTCGAGTTCTCTTCCTTCTTCAGGAGCTTTTTG<br>2. CCGGCCTGGCAGAAAGATTCCACATCTCGAGATGTGGAATCTTTCTGCCAGGTTTTTG<br>3. CCGGGCTCCTGAAGAAGGAAGAGAACTCGAGTTCTCTTCCTTCTTCAGGAGCTTTTTG
PDIA6	1. CCGGCCATCGAATTTCAACCGAGAACTCGAGTTCTCGGTTGAAATTCGATGGTTTTTG<br>2. CCGGGAGATTATCAACGAGGACATTCTCGAGAATGTCCTCGTTGATAATCTCTTTTTG

	3. CCGGGCAGATAAGCATCATTCCCTACTCGAGTAGGGAATGATGC TTATCTGCTTTTTG

Quantitative RT-PCR

The relative mRNA expression levels of target genes were measured using quantitative RT-PCR. Cells were treated as described at 37°C, harvested by trypsinization, washed with Dulbecco's phosphate-buffered saline (GIBCO), and then RNA was extracted using the RNeasy Mini Kit (QIAGEN). qPCR reactions were performed on cDNA prepared from 500 ng of total cellular RNA using the QuantiTect Reverse Transcription Kit (QIAGEN). The FastStart Universal SYBR Green Master Mix (Roche), cDNA, and appropriate primers purchased from Integrated DNA Technologies (Table S2) were used for amplifications (6 min at 95°C then 45 cycles of 10 s at 95°C, 30 s at 60°C) in an ABI 7900HT Fast Real Time PCR machine. Primer integrity was assessed by a thermal melt to confirm homogeneity and the absence of primer dimers. Transcripts were normalized to the housekeeping genes Rplp2 and all measurements were performed in triplicate. Data were analyzed using the RQ Manager and DataAssist 2.0 softwares (ABI). qPCR data are reported as mean ± 95% confidence interval as calculated in DataAssist 2.0.

Table S2: Sequences of Primers for qPCR.

Gene	Forward Primer	Reverse Primer
HspA5 (BiP)	GCCTGTATTTCTAGACCTGCC	TTCATCTTGCCAGCCAGTTG
PDIA1	CGAGTTCACCGAGCAGACAG	CAGGATCTTGCCCTTGAAGC
PDIA3	ATGCCCTAAGGATGGGTTCC	CCCAGCAGTGTCAGCAATTC
PDIA4	AGTGGGGAGGATGTCAATGC	TGGCTGGGATTTGATGACTG
PDIA5	AAGTTGACCTGAGCCCGAAG	CCAGGATCTTCCTCCCACAG
PDIA6	CGGAGCGGAGGATACAGTTC	GCAGTGTCCACACCAAGGAG
Rplp2	CGTCGCCTCCTACCTGCT	CATTCAGCTCACTGATAACCTTG

Synthesis and Characterization

Flash column chromatography was carried out using a standard setup. ^{1}H NMR spectra were recorded on a Varian INOVA-400 400MHz spectrometer. Chemical shifts are reported in δ units (ppm) relative to residual solvent peak. Coupling constants (*J*) are reported in hertz (Hz). Characterization data are reported as follows: chemical shift, multiplicity (s=singlet, d=doublet, t=triplet, q=quartet, br=broad, m=multiplet), coupling constants, number of protons, mass to charge ratio. The compound's identity was confirmed via high-resolution mass spectrometry.

Waters Micromass ZQ/Waters 2795 Separation Module/Waters 2996 Photodiode Array Detector/Waters 2424 Evaporative Light Scattering Detector system. Separations were carried out on an XTerra MS C_{18} 5 µm 4.6 × 50 mm column at ambient temperature using a mobile phase of water-methanol containing 0.1% formic acid.

AA147

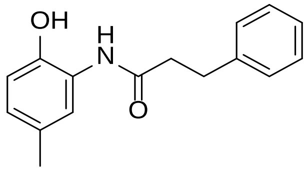

265 mg (2.1 mmol) of 2-amino-*p*-cresol was added to 160 µL (1 mmol) of hydrocinnamoyl chloride with 200 µL (1.5 mmol) of trimethylamine (TEA) in 2.5 mL of methylene chloride (DCM) on ice. The reaction was allowed to warm to room temperature over 16 hr. The reaction was diluted with DCM and quenched with 1 M HCl. The reaction was washed once more with 1 M HCl followed by a wash with brine. The organic layer was dried with magnesium sulfate, filtered, and concentrated and purified with 4:1 hexane:ethyl acetate. 217 mg was obtained giving 85% yield.

^{1}H NMR (400 MHz, Chloroform-*d*) δ 8.41 (s, 1H), 7.32 (t, *J* = 7.2 Hz, 2H), 7.24 (d, *J* = 7.7 Hz, 3H), 6.91 (d, *J* = 7.4 Hz, 2H), 6.60 (d, *J* = 7.4 Hz, 1H), 3.07 (t, *J* = 7.5 Hz, 2H), 2.75 (q, *J* = 8.1, 7.2 Hz, 2H), 2.23 (d, *J* = 7.1 Hz, 3H). M/Z=256.1334

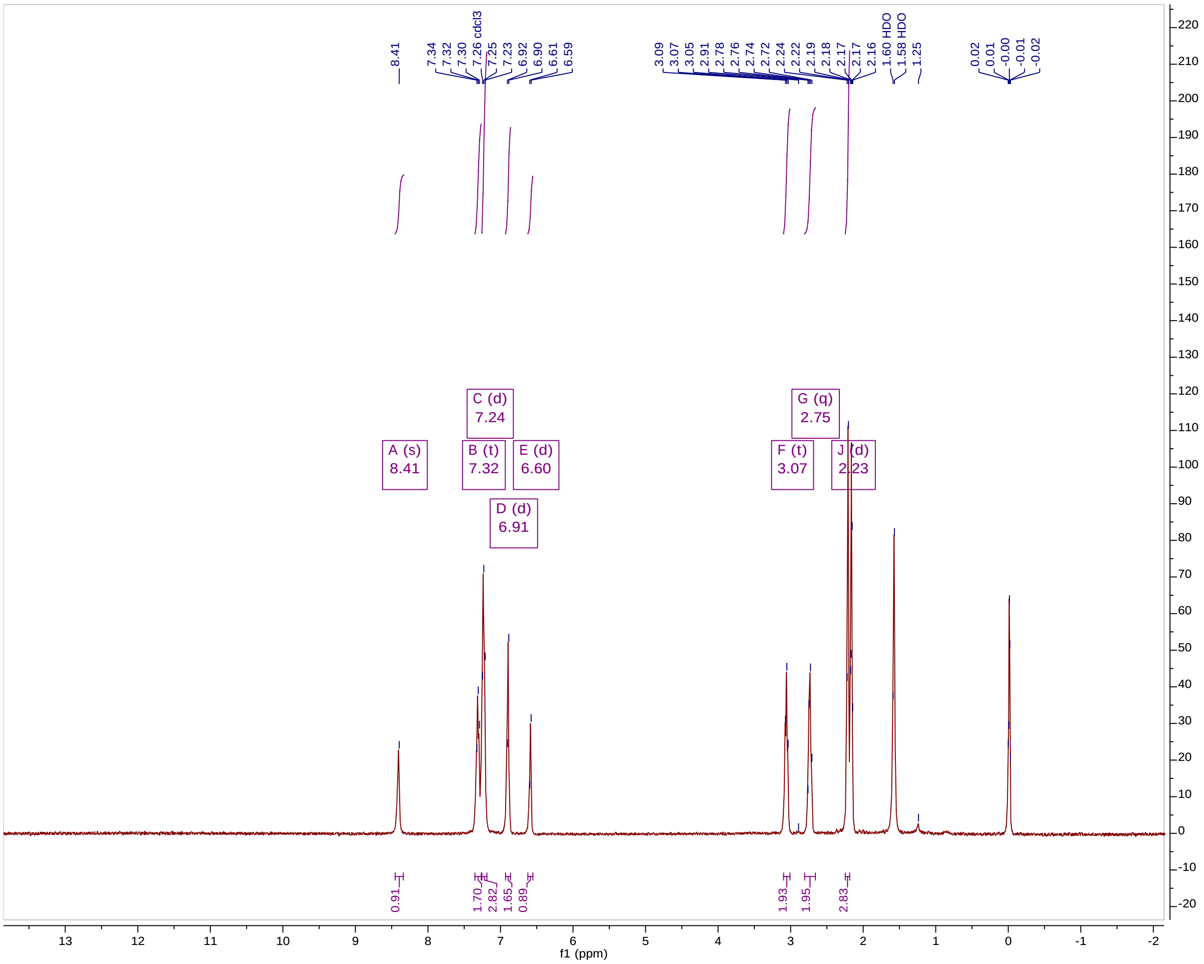

AA147-1

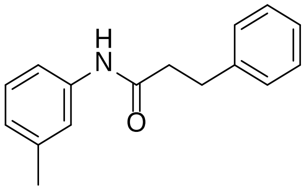

110 µL of *m*-toluidine (1 mmol) was added to 150 µL of hydrocinnamoyl chloride (1 mmol) with 140 µL of TEA in DCM on ice. The reaction proceeded to room temperature over 16 hr where it was then diluted with DCM and washed twice with 1 M HCL, followed by a wash with brine. The reaction was concentrated and purified via column chromatography using 4:1 hexane:ethyl acetate giving a 89% yield.

^{1}H NMR (400 MHz, Chloroform-*d*) δ 7.30 (d, *J* = 7.3 Hz, 3H), 7.25 (d, *J* = 9.1 Hz, 4H), 7.18 (d, *J* = 4.7 Hz, 2H), 6.91 (s, 1H), 3.06 (t, *J* = 7.6 Hz, 2H), 2.65 (t, *J* = 7.6 Hz, 2H), 2.33 (s, 3H). M/Z=240.1389

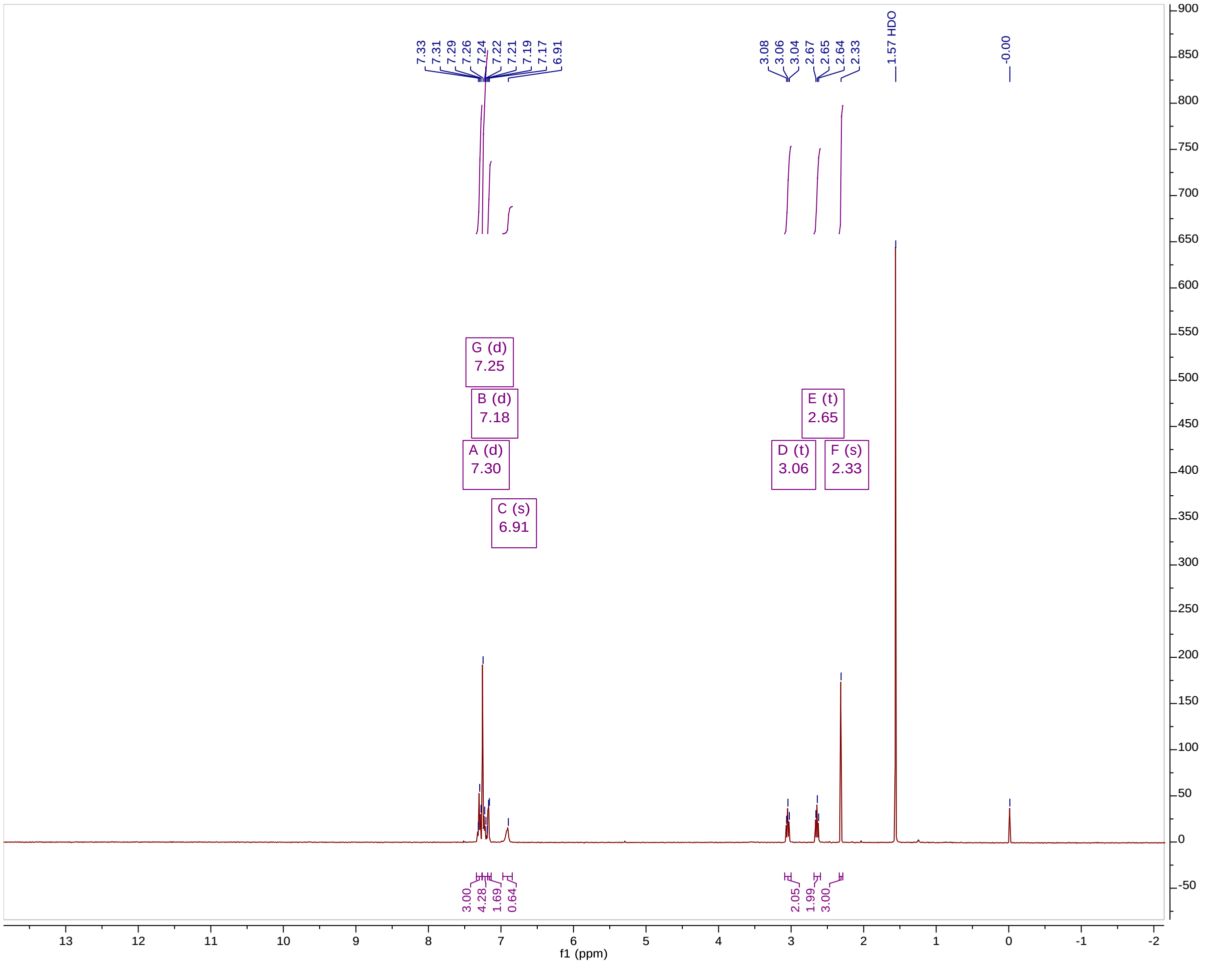

AA147-2

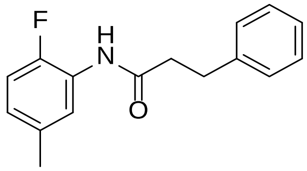

137 mg (1.1 mmol) of 3-fluoro-4-methylaniline was mixed with 150 µL (1 mmol) of hydrocinnamoyl chloride and 140 µL of TEA in DCM on ice, warming to room temperature over 16 hr. The reaction was worked up and purified as described above to give 84% yield.

^{1}H NMR (400 MHz, Chloroform-*d*) δ 8.14 (d, *J* = 7.6 Hz, 1H), 7.35 – 7.28 (m, 2H), 7.23 (dd, *J* = 15.9, 7.7 Hz, 4H), 6.93 (dd, *J* = 10.9, 8.3 Hz, 1H), 6.82 (d, *J* = 6.3 Hz, 1H), 3.07 (t, *J* = 7.7 Hz, 2H), 2.71 (t, *J* = 7.7 Hz, 2H), 2.32 (s, 3H). M/Z=258.1295

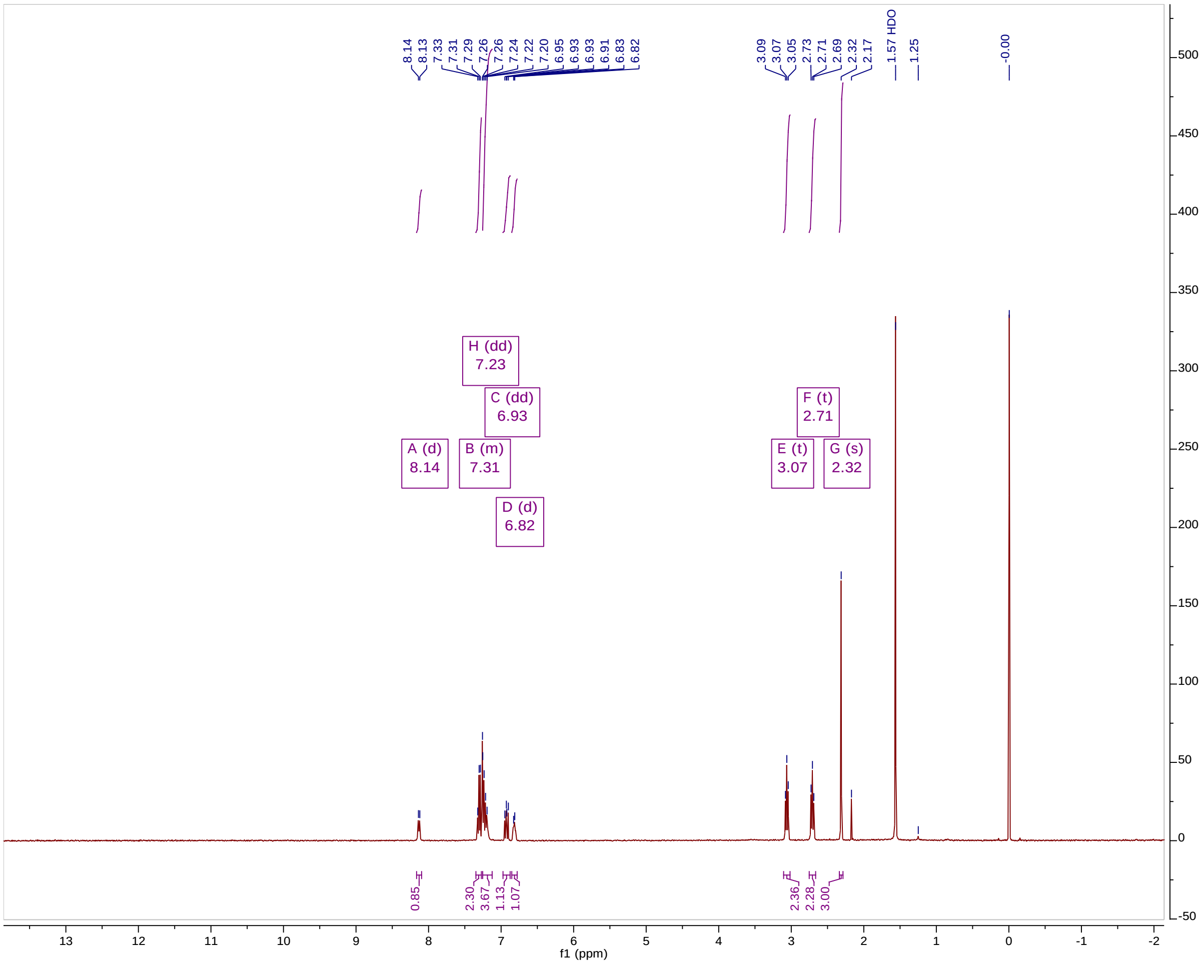

AA147-3

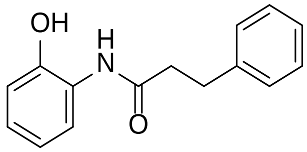

218 mg of 2-aminophenol (2 mmol) was added to 150 µL of hydrocinnamoyl chloride with 140 µL of TEA in DCM on ice. The reaction proceeded for 16 hours warming to room temperature. The reaction was worked up and purified as described above to give 37% yield.

^{1}H NMR (400 MHz, Chloroform-*d*) δ 8.68 (s, 1H), 7.56 – 6.93 (m, 8H), 6.79 (d, *J* = 23.5 Hz, 2H), 3.09 (s, 2H), 2.77 (s, 2H). M/Z=242.1178

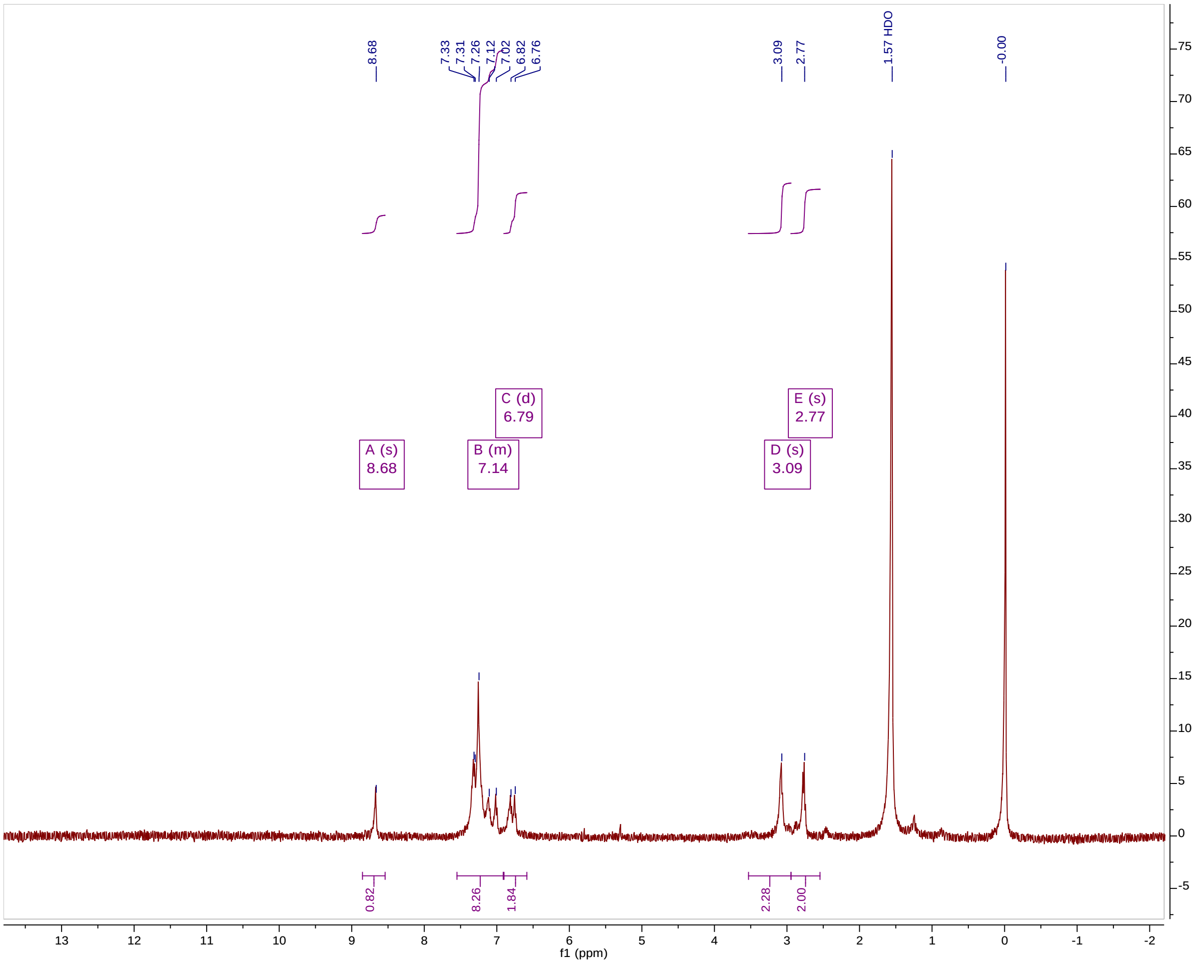

AA147-4

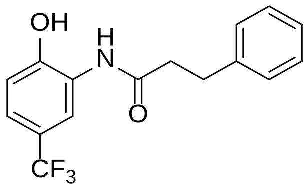

87 mg (0.5 mmol) of 2-amino-5-(trifluoromethyl)phenol were mixed with 150 µL (1 mmol) of hydrocinnamoyl chloride with 160 µL (2 mmol) of pyridine in DCM on ice for 16 h. The reaction was diluted in DCM and washed twice with 1M HCL followed by a wash with brine. The organic layer was collected, concentrated, and purified via column chromatography using 6:1 toluene ethyl acetate giving 59% yield.

^{1}H NMR (400 MHz, Chloroform-*d*) δ 9.19 (s, 1H), 7.34 (t, *J* = 8.1 Hz, 3H), 7.26 (dd, *J* = 14.8, 7.0 Hz, 2H), 7.16 (s, 1H), 7.06 (d, *J* = 8.6 Hz, 1H), 6.98 (s, 1H), 3.09 (t, *J* = 7.4 Hz, 2H), 2.79 (t, *J* = 7.4 Hz, 2H). M/Z=310.1051

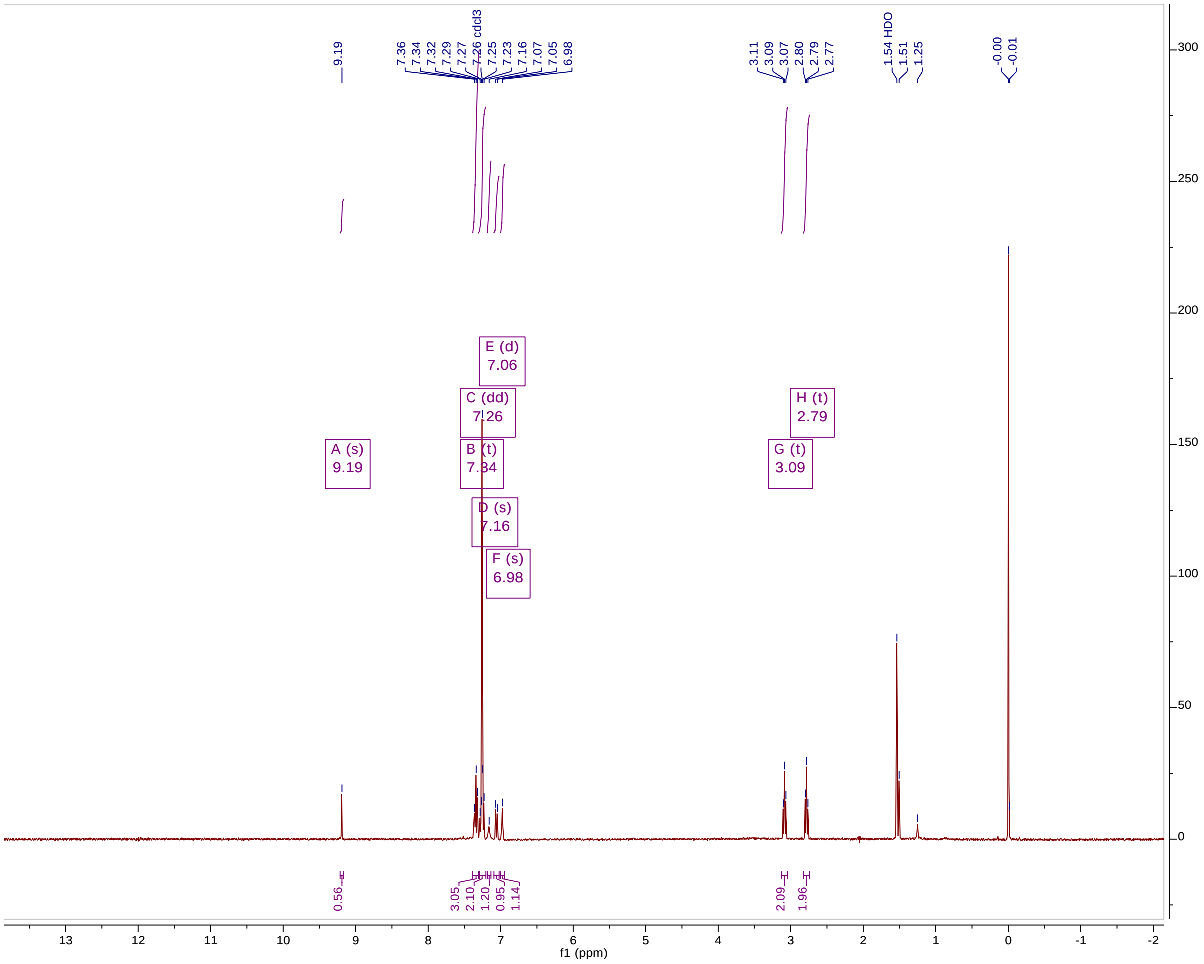

AA147-5

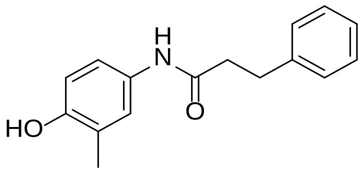

370 mg (3 mmol) of 4-amino-*o*-cresol was added to 150 µL of hydrocinnamoyl chloride (1 mmol) with 140 µL (1 mmol) of TEA in DCM on ice for 16 hours. The reaction was diluted with DCM and washed twice with 1 M HCL and once with brine. The reaction was concentrated and purified via column chromatography using 4:1 hexanes ethyl acetate to give 41% yield.

^{1}H NMR (400 MHz, Chloroform-*d*) δ 8.35 (s, 1H), 7.59 – 7.52 (m, 1H), 7.34 – 7.20 (m, 3H), 7.10 (td, *J* = 7.6, 2.0 Hz, 1H), 6.95 – 6.85 (m, 2H), 6.70 (s, 1H), 3.18 (t, *J* = 7.6 Hz, 2H), 2.76 (t, *J* = 7.6 Hz, 2H), 2.23 (s, 3H). M/Z=256.1331

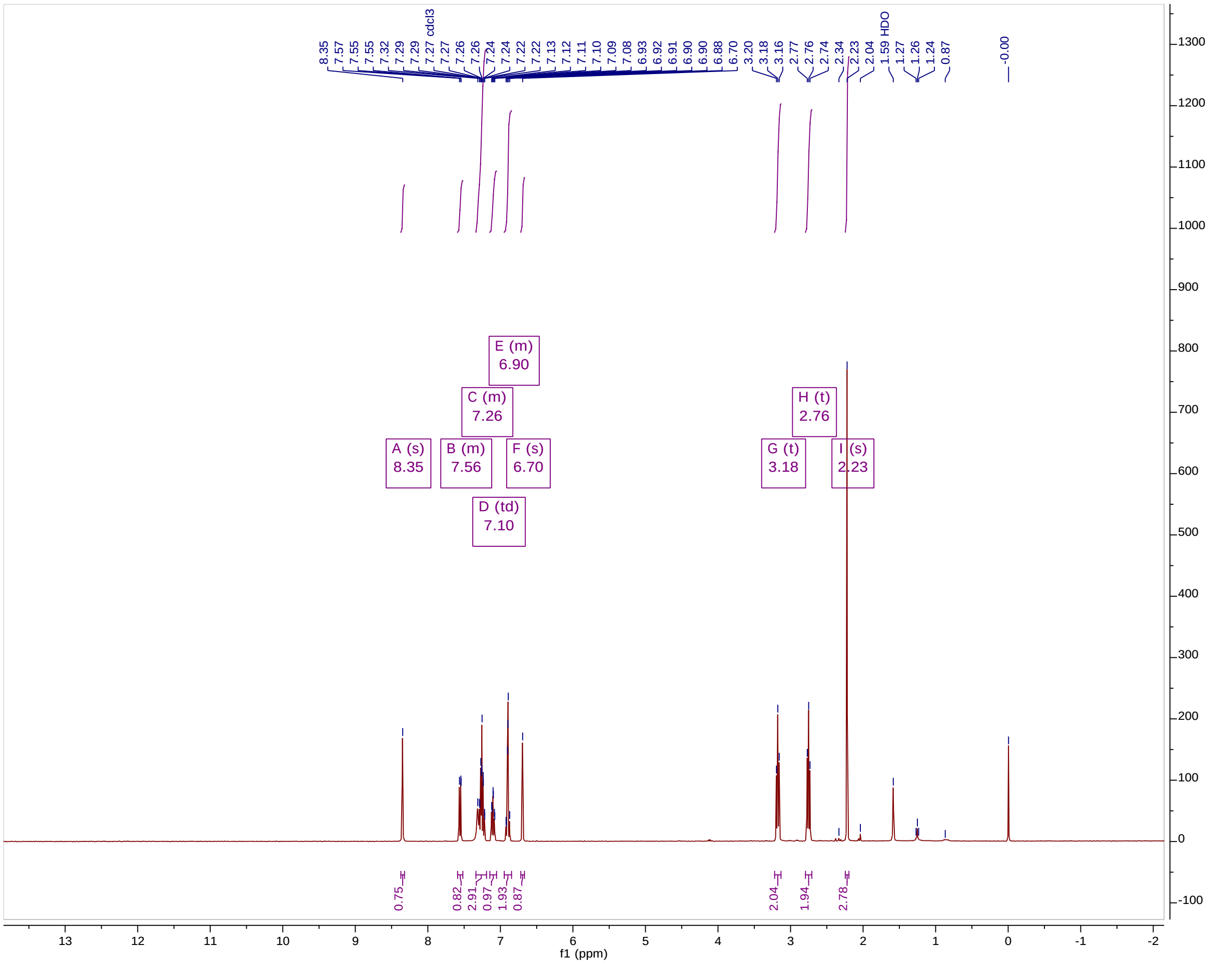

AA147-6

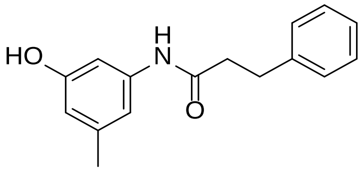

160 mg of 5-amino-*m*-cresol was weighed and added to 150 µL of hydrocinnamoyl chloride with 150 µL of TEA on ice for 16 hours. The reaction was worked up as described above to give 38% yield.

^{1}H NMR (400 MHz, Chloroform-*d*) δ 7.35 – 7.26 (m, 2H), 7.24 (d, J = 9.2 Hz, 4H), 6.91 (s, 1H), 6.52 (s, 1H), 6.43 (s, 1H), 5.62 (s, 1H), 3.05 (t, J = 7.6 Hz, 2H), 2.65 (t, J = 7.6 Hz, 2H), 2.25 (s, 3H). M/Z=256.1340

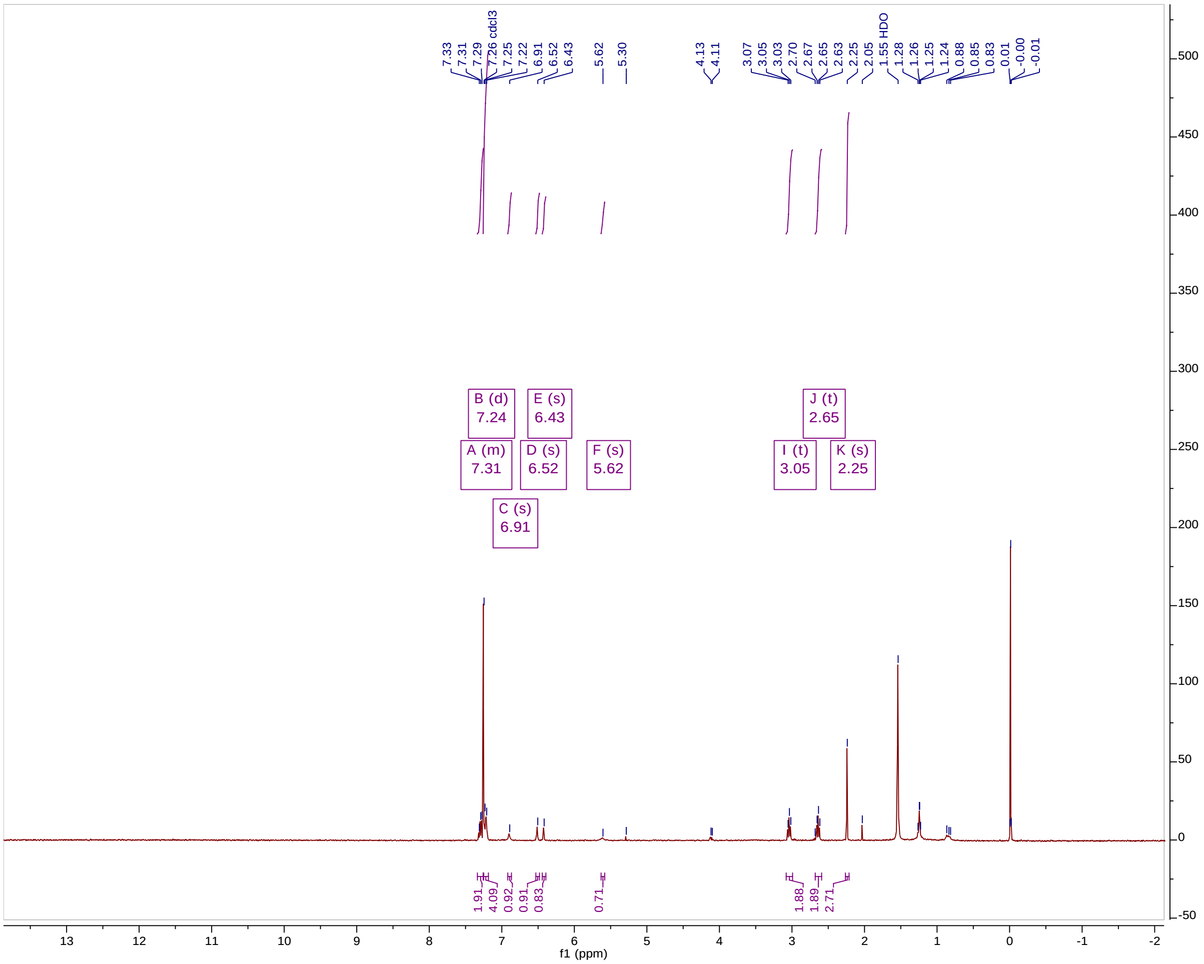

AA147-7

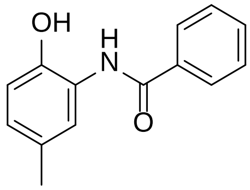

122 mg (1 mmol) of benzoic acid was dissolved in 3 mL of DCM. 180 µL (2.1 mmol) of oxalyl chloride was added with a few drops of DMF for 3 hours at room temperature. The reaction was concentrated under reduced pressure and dissolved in DCM. 185 mg (1.5 mmol) of 2-amino-*p*-cresol was added with 210 µL (1.5 mM) of TEA for 16 hours on ice rooming to room temperature. The reaction was diluted with DCM and washed with 1 M HCl followed by a wash with brine. The reaction was dried with $MgSO_4$ and concentrated and purified via column chromatography with 4:1 hexane ethyl acetate to give 52% yield.

^{1}H NMR (400 MHz, Chloroform-*d*) δ 8.36 (s, 1H), 8.04 (s, 1H), 7.94 – 7.87 (m, 2H), 7.61 (t, *J* = 7.3 Hz, 1H), 7.53 (t, *J* = 7.5 Hz, 2H), 6.99 (d, *J* = 8.7 Hz, 3H), 2.30 (s, 3H). M/Z=228.1024

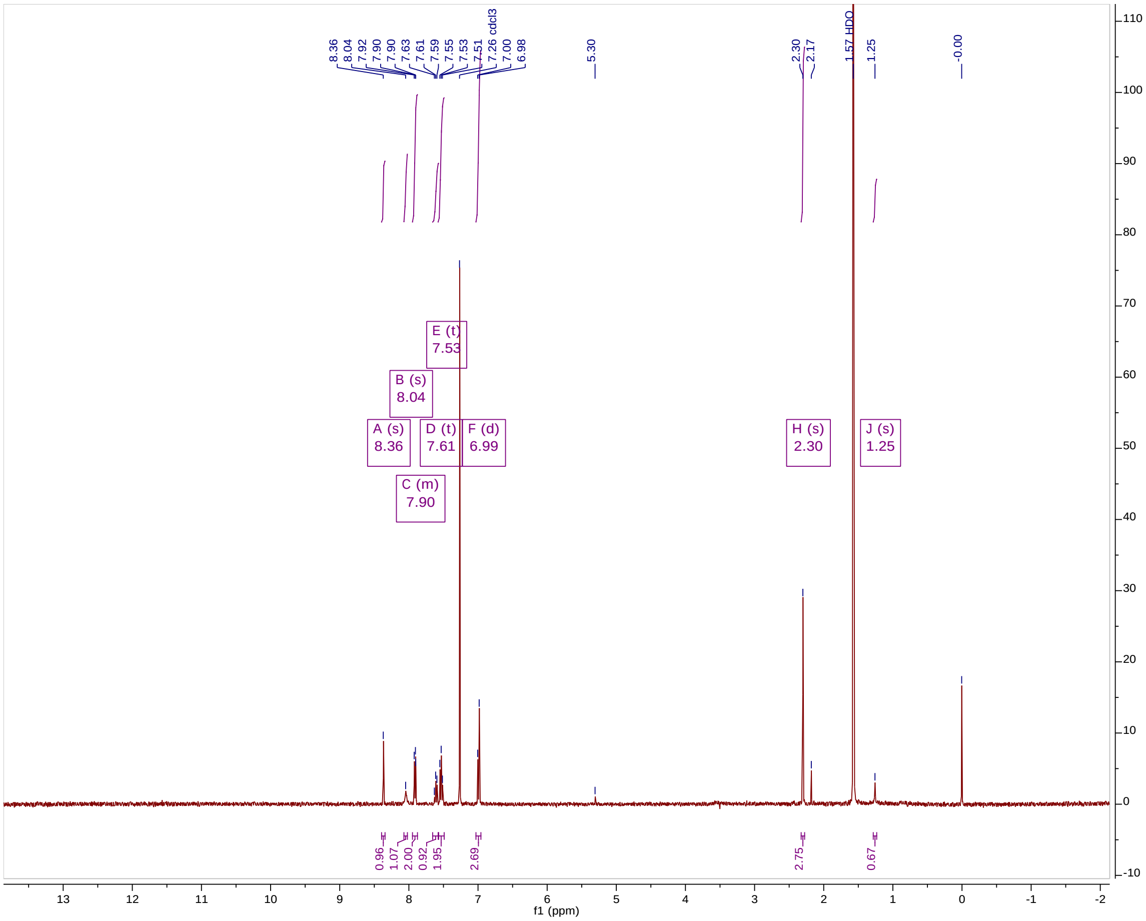

AA147-8

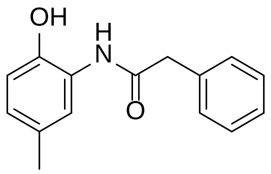

180 µL of oxalyl chloride (2.1 mmol) was mixed with 134.1 mg of 2-phenylacetic acid (1 mmol) with a catalytic amount of dimethylformamide in 3 mL of methylene chloride at room temperature for 2 hours. The resulting mixture was concentrated and reconstituted in 2.5 mL of DCM. 183.3 mg of 2-amino-*p*-cresol (1.5 mmol) and 200 µL of trimethylamine (1.5 mmol) was added to the reaction on ice warming to room temperature for 16 hours. The reaction was washed twice with 1M HCL followed by a wash with brine. The organic layer was concentrated and purified via column chromatography with 4:1 hexane ethyl acetate. 82 mg of product was obtained giving 34% yield.

H^1 NMR (400 MHz, Chloroform-*d*) δ 8.43(s, 1H), 7.49-7.32 (m, 5H), 7.26(s, 1H), 6.89 (s, 2H), 6.56(s, 1H), 3.81 (s, 2H), 2.2 (s, 3H). M/Z=242.1177

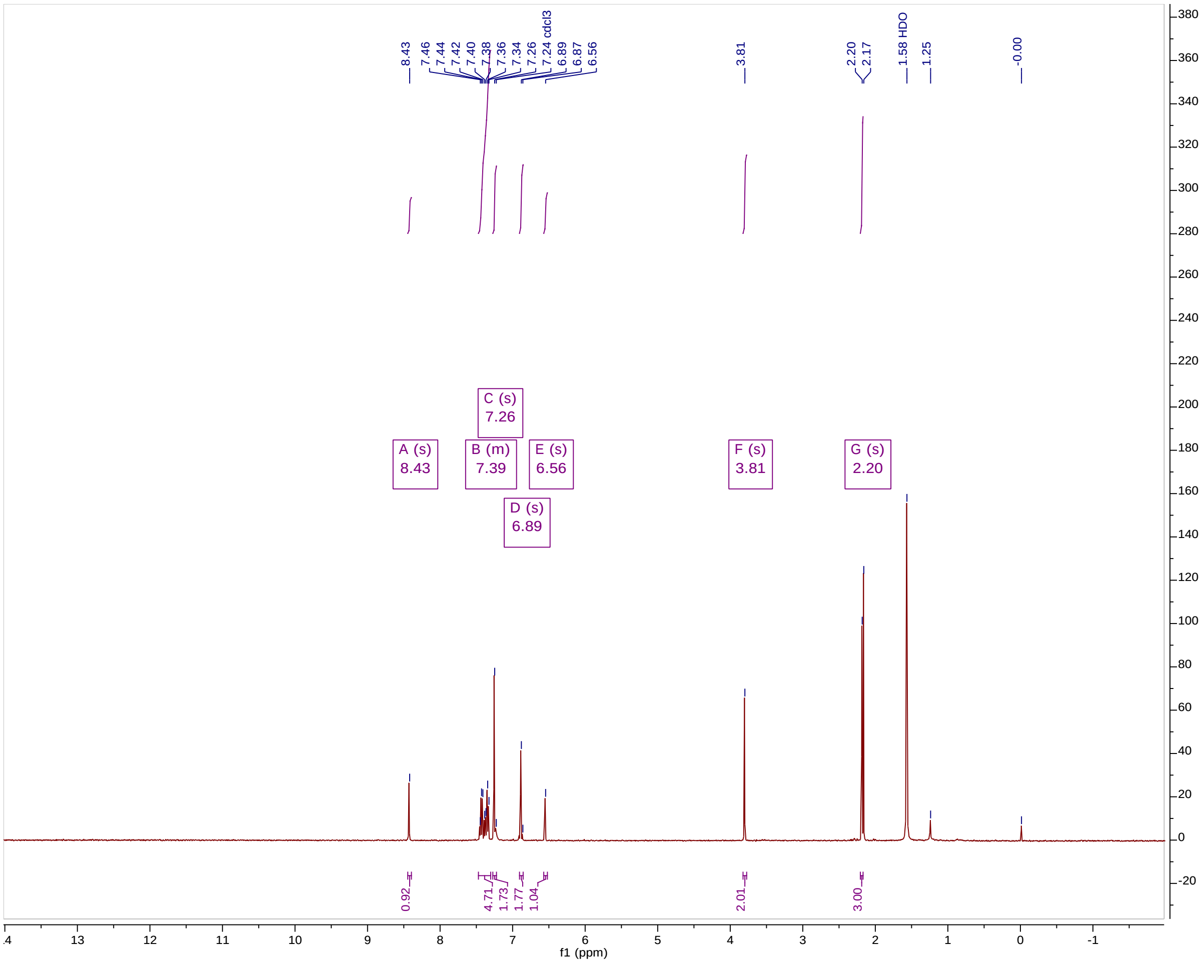

AA147-9

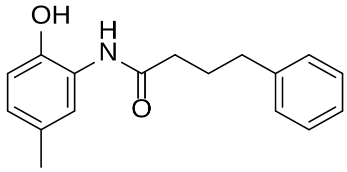

166 mg (1 mmol) of 4-phenylbutyric acid was added to 180 µL of oxalyl chloride in DCM with a few drops of DMF for 3 hours. The reaction was concentrated at low pressure and dissolved in DCM. 186 mg (1.5 mmol) of 2-amino-*p*-cresol was added to the reaction mixture with 210 µL (1.5 mmol) of TEA on ice for 16 hours. The reaction was washed with 1M HCl followed by a wash with brine. The reaction was dried over magnesium sulfate and concentrated and purified via column chromatography using 4:1 hexane ethyl acetate giving 60% yield.

^{1}H NMR (400 MHz, Chloroform-*d*) δ 8.49 (s, 1H), 7.31 (t, *J* = 7.4 Hz, 3H), 7.21 (d, *J* = 7.8 Hz, 3H), 6.92 (s, 2H), 6.74 (s, 1H), 2.73 (t, *J* = 7.4 Hz, 2H), 2.43 (t, *J* = 7.4 Hz, 2H), 2.25 (s, 3H), 2.11 (q, *J* = 7.5 Hz, 2H). M/Z=270.1494

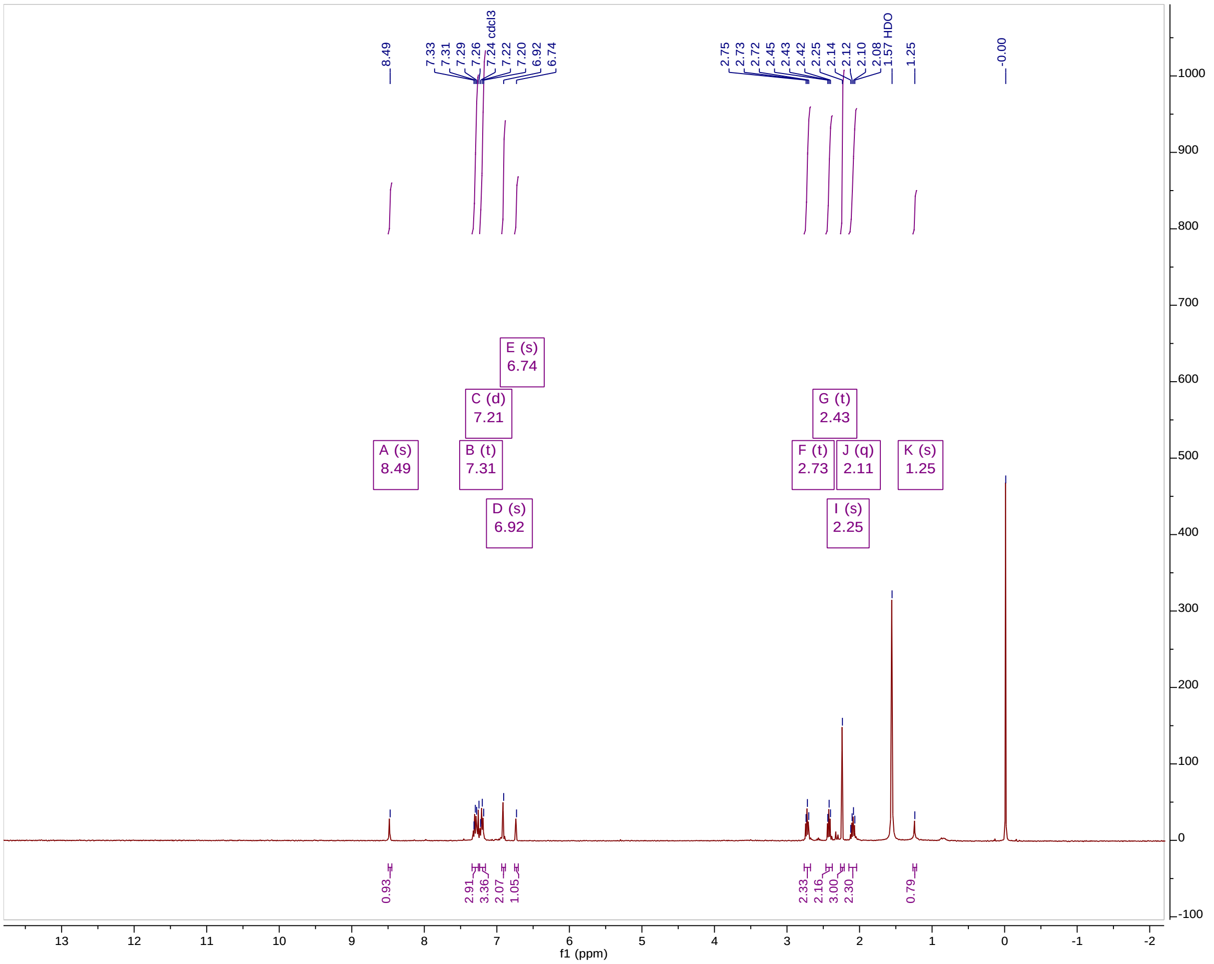

AA147-10

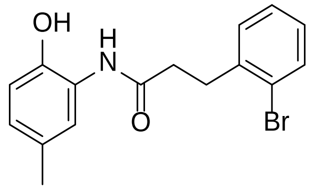

255 mg (1 mmol) of 3-(2-bromophenyl)propionic acid was dissolved in 3 mL of DCM, 180 µL (2.1 mmol) of oxalyl chloride was added with a few drops of DMF. The acyl halide was concentrated and dissolved in 3 mL of DCM, 254 mg of 2-amino-*p*-cresol was added with 160 µL of pyridine on ice rooming to room temperature for 16 hours. The reaction was diluted with DCM and washed once with 1 M HCl followed by a wash with brine. The reaction was dried and concentrated and purified via column chromatography using 4:1 hexane ethyl acetate giving a 41% yield.

^{1}H NMR (400 MHz, Chloroform-*d*) δ 8.35 (s, 1H), 7.59 – 7.52 (m, 1H), 7.34 – 7.20 (m, 3H), 7.10 (td, *J* = 7.6, 2.0 Hz, 1H), 6.95 – 6.85 (m, 2H), 6.70 (s, 1H), 3.18 (t, *J* = 7.6 Hz, 2H), 2.76 (t, *J* = 7.6 Hz, 2H), 2.23 (s, 3H). M/Z= M/Z=334.0429

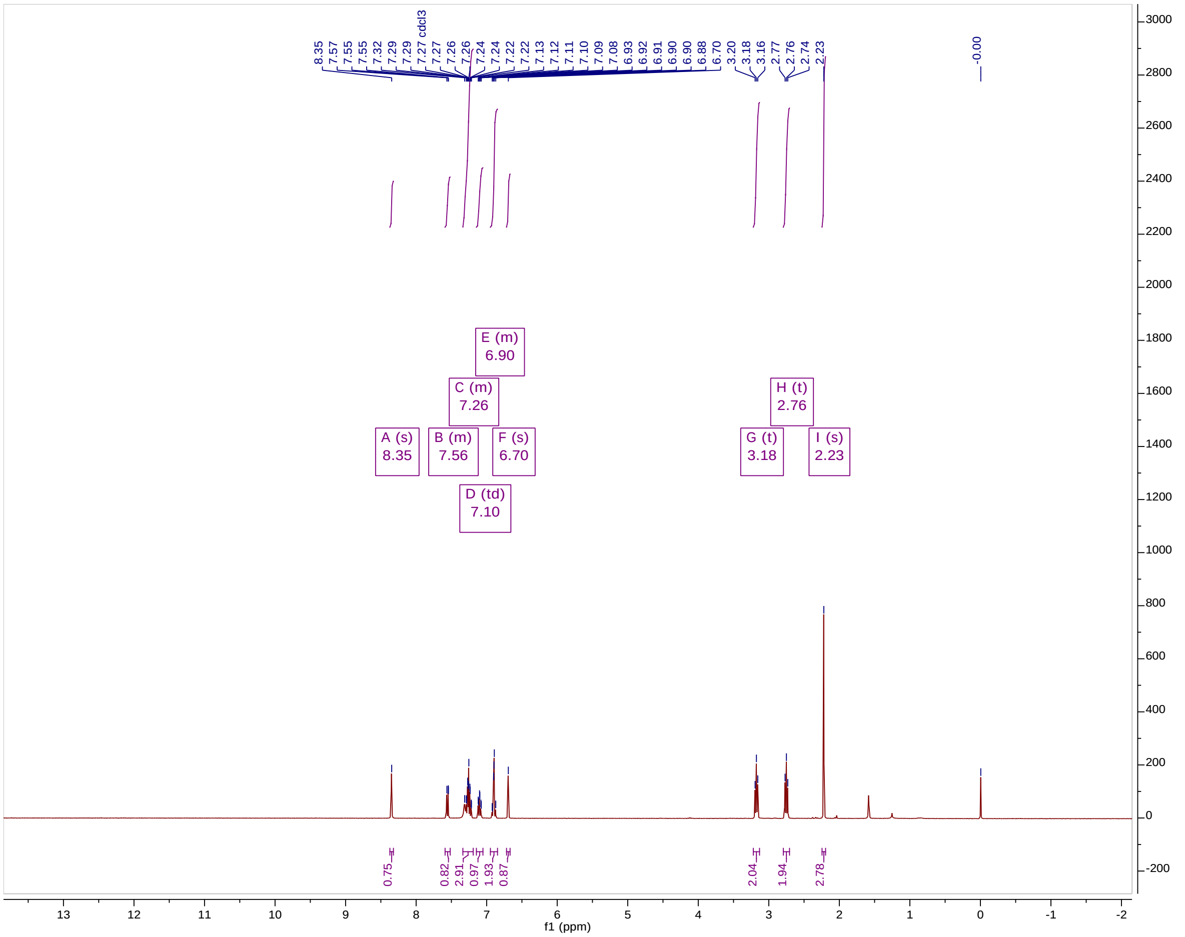

AA147-11

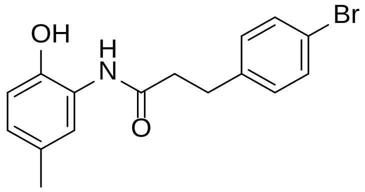

230 mg of 3-(4-bromophenyl)propionic acid was mixed with 180 µL of oxalyl chloride with a catalytic amount of DMF in DCM for 3 hours. The reaction was concentrated under reduced pressure and dissolved in 3 mL of DCM. 280 mg of 2-amino-*p*-cresol and 140 µL of TEA were added on ice. The reaction was allowed to warm to room temperature over 16 hours. The reaction was diluted in DCM and washed once with HCl followed by a wash with brine. The reaction was dried with magnesium sulfate and concentrated and purified via column chromatography using 4:1 hexane ethyl acetate to give a 45% yield.

1H NMR (400 MHz, Chloroform-d) δ 8.22 (s, 1H), 7.47 – 7.37 (m, 3H), 7.18 (s, 1H), 7.12 (dd, J = 8.3, 4.4 Hz, 2H), 6.96 – 6.84 (m, 2H), 6.66 (d, J = 1.8 Hz, 1H), 3.03 (t, J = 7.4 Hz, 2H), 2.71 (t, J = 7.5 Hz, 2H), 2.24 (s, 3H). M/Z=334.0443

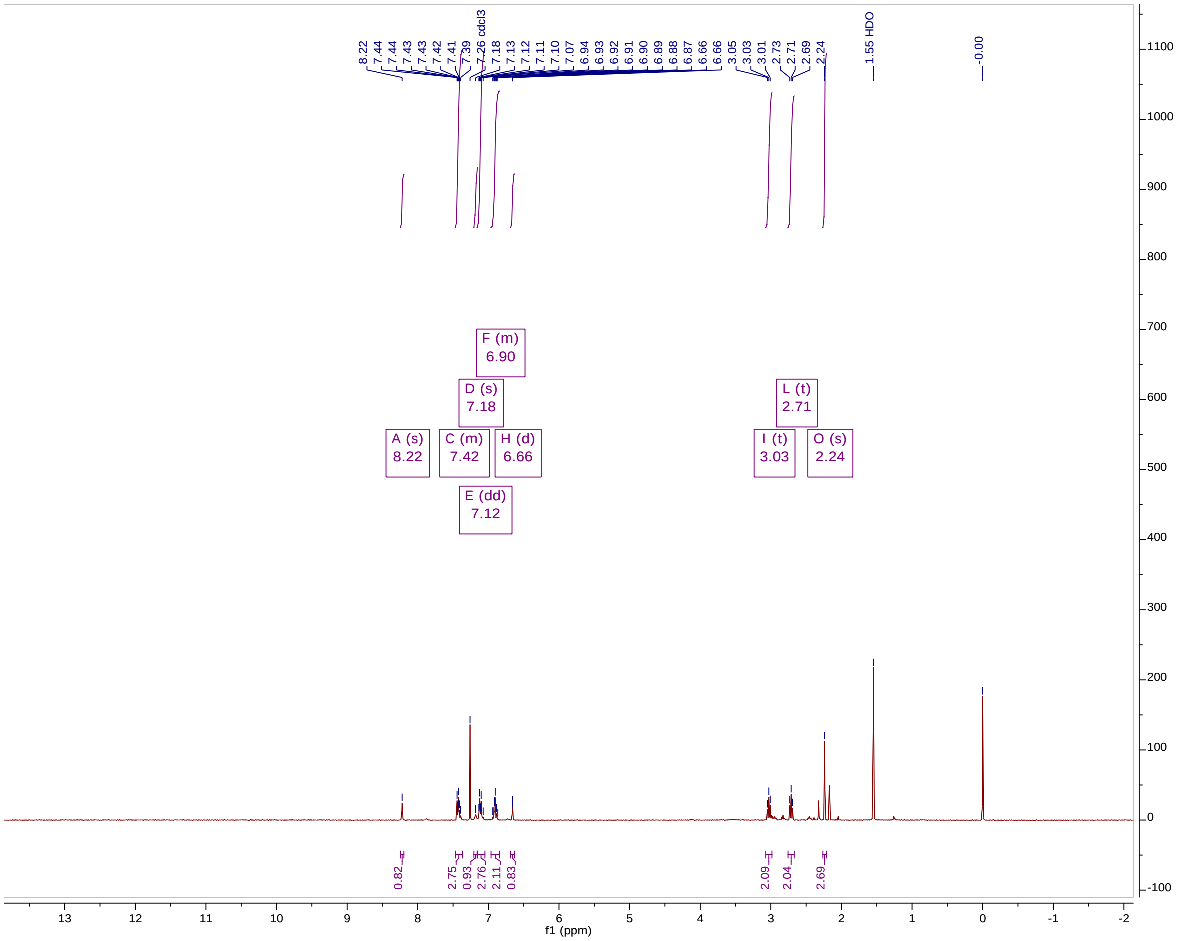

AA147-12

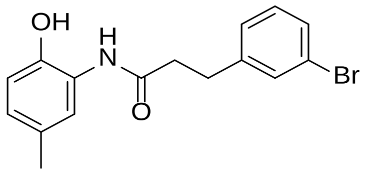

230 mg of 3-(3-bromophenyl)propionic acid was mixed with 180 µL of oxalyl chloride with a catalytic amount of DMF in DCM for 3 hours. The reaction was concentrated under reduced pressure and dissolved in 3 mL of DCM. 280 mg of 2-amino-*p*-cresol and 140 µL of TEA were added on ice. The reaction was allowed to warm to room temperature over 16 hours. The reaction was diluted in DCM and washed once with HCl followed by a wash with brine. The reaction was dried with magnesium sulfate and concentrated and purified via column chromatography using 4:1 hexane ethyl acetate to give a 41% yield.

1H NMR (400 MHz, Chloroform-d) δ 8.34 (s, 1H), 7.52 (s, 1H), 7.38 (dd, J = 5.7, 2.1 Hz, 2H), 7.23 – 7.08 (m, 2H), 6.97 – 6.85 (m, 2H), 6.80 – 6.75 (m, 1H), 3.03 (t, J = 7.6 Hz, 2H), 2.71 (t, J = 7.6 Hz, 2H), 2.25 (s, 3H). M/Z=334.0435

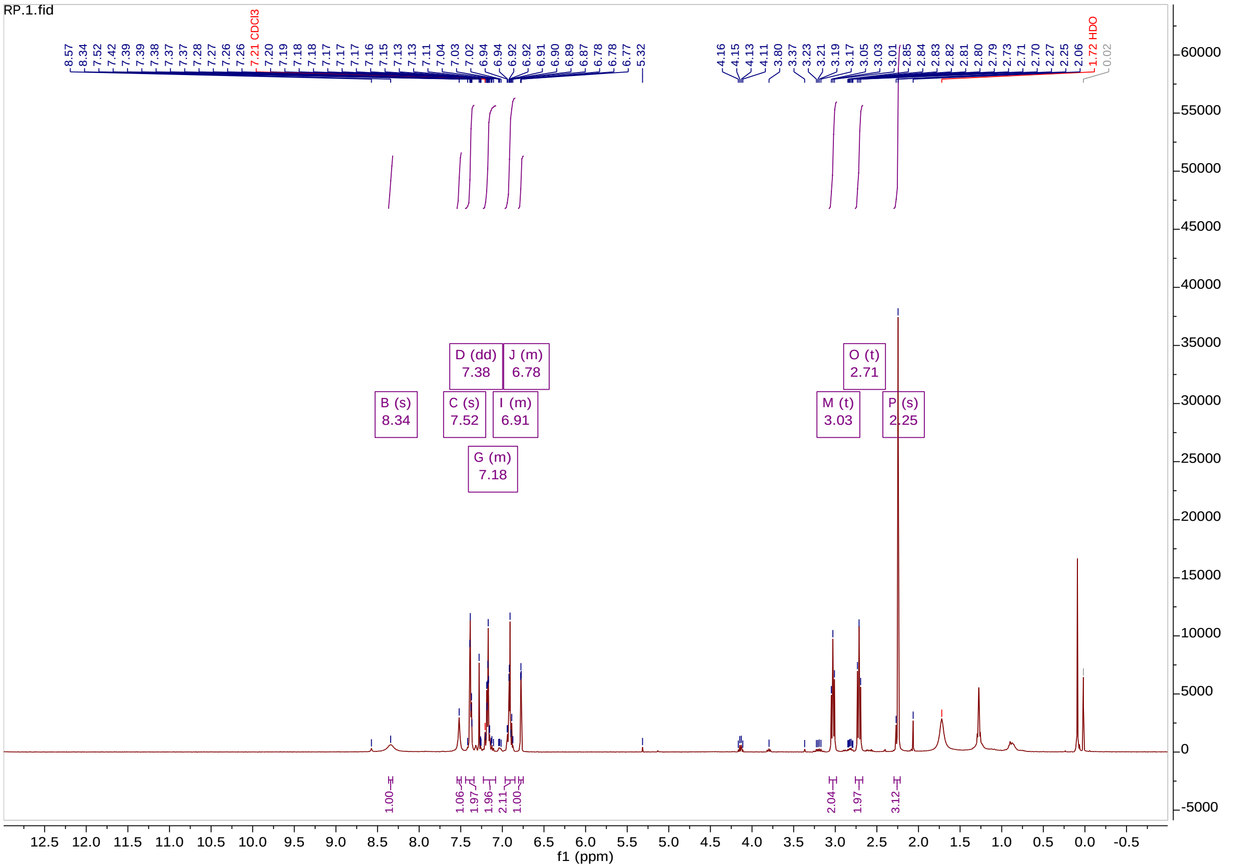

AA147-13

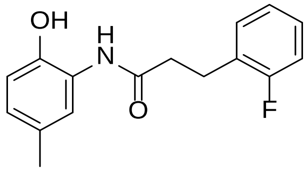

165 mg of 3-(2-fluorophenyl)propionic acid was mixed with 180 µL of oxalyl chloride with a catalytic amount of DMF in DCM for 3 hours. The reaction was concentrated under reduced pressure and dissolved in 3 mL of DCM. 290 mg of 2-amino-*p*-cresol and 200 µL of TEA were added on ice. The reaction was allowed to warm to room temperature over 16 hours. The reaction was diluted in DCM and washed twice with 1 M HCl followed by a wash with brine. The reaction was dried with magnesium sulfate and concentrated and purified via column chromatography using 4:1 hexane ethyl acetate to give 256 mg 93% yield.

^{1}H NMR (400 MHz, Chloroform-*d*) δ 8.35 (s, 1H), 7.51 (s, 1H), 7.30 – 7.19 (m, 1H), 6.98 (d, *J* = 7.6 Hz, 1H), 6.96 – 6.83 (m, 4H), 6.75 (d, *J* = 1.7 Hz, 1H), 3.02 (t, *J* = 7.6 Hz, 2H), 2.70 (t, *J* = 7.5 Hz, 2H), 2.21 (s, 3H). M/Z=274.1244

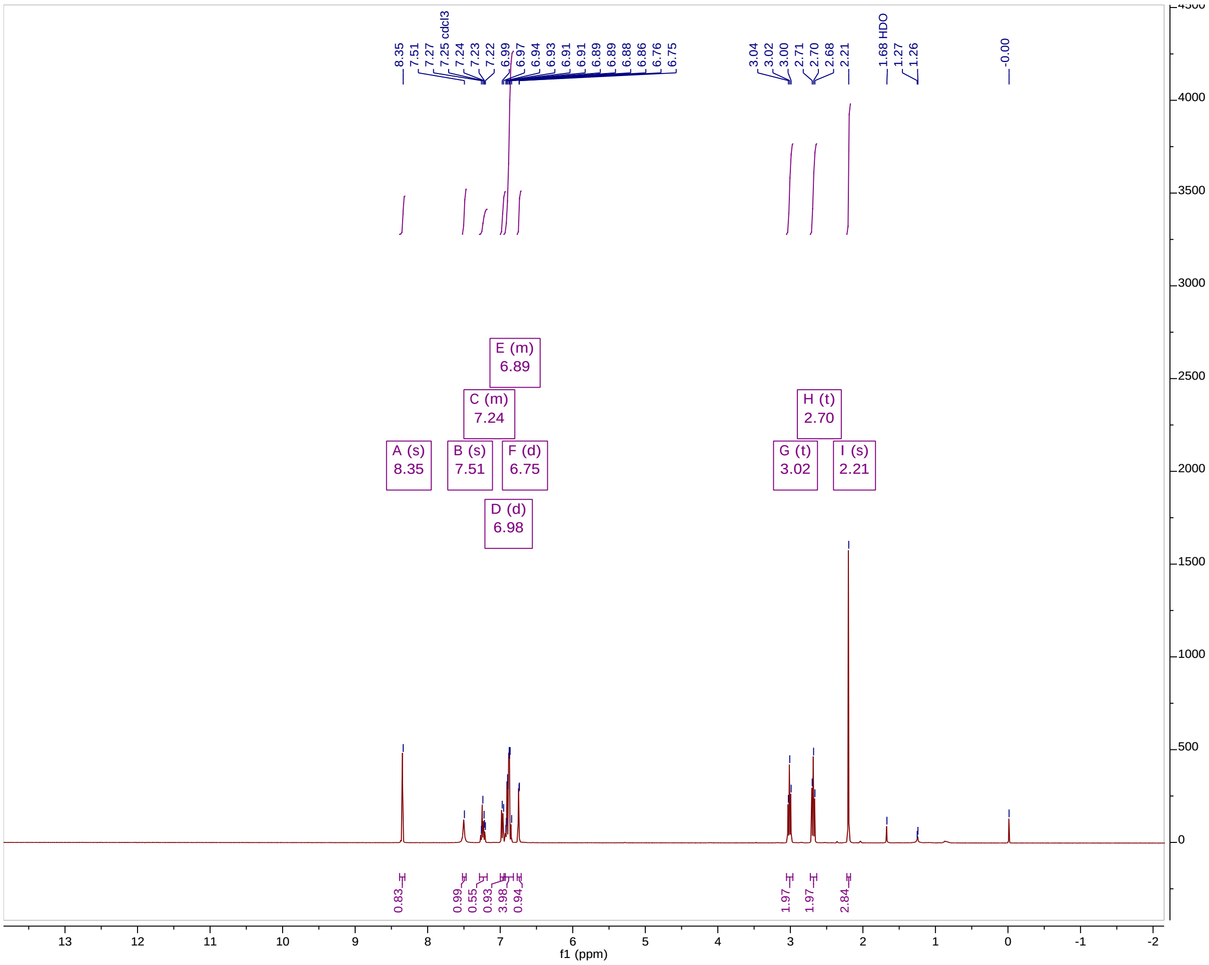

AA147-14

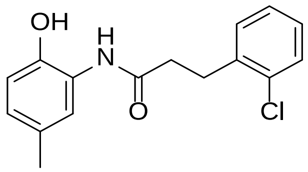

186 mg of 3-(2-chlorophenyl)propionic acid was mixed with 180 µL of oxalyl chloride with a catalytic amount of DMF in DCM for 3 hours. The reaction was concentrated under reduced pressure and dissolved in 3 mL of DCM. 270 mg of 2-amino-*p*-cresol and 300 µL of pyridine were added on ice. The reaction was allowed to warm to room temperature over 16 hours. The reaction was diluted in DCM and washed twice with 1 M HCl followed by a wash with brine. The reaction was dried with magnesium sulfate and concentrated and purified via column chromatography using 9:1 toluene ethyl acetate to give 61 mg 21% yield.

^{1}H NMR (400 MHz, Chloroform-*d*) δ 8.38 (s, 1H), 7.43 – 7.33 (m, 2H), 7.34 – 7.09 (m, 3H), 6.95 – 6.79 (m, 2H), 6.72 (s, 1H), 3.16 (t, *J* = 7.6 Hz, 2H), 2.74 (t, *J* = 7.6 Hz, 2H), 2.22 (s, 3H), 1.26 (s, 2H). M/Z=290.0950

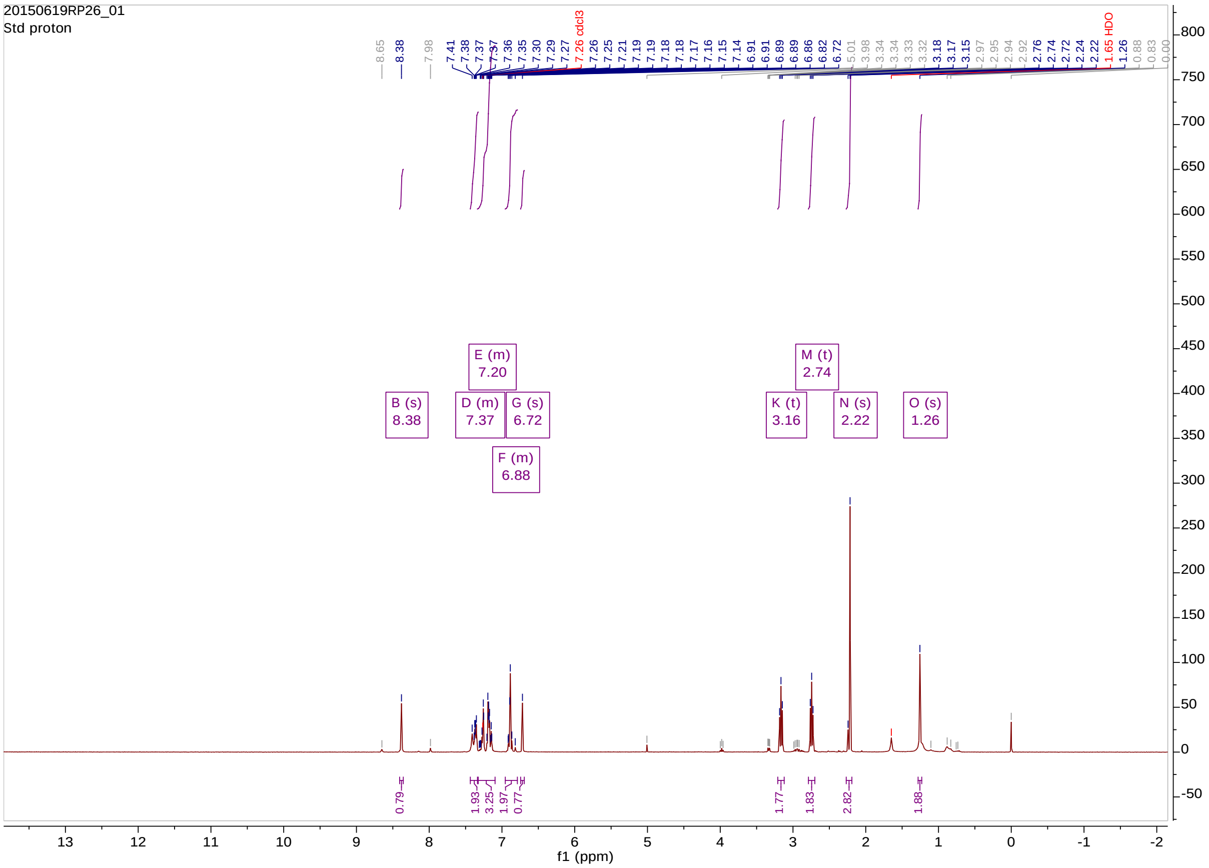

AA147-15

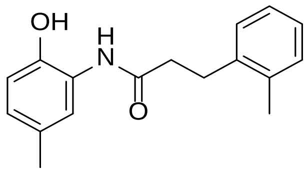

165 mg of 3-(2-methylphenyl)propionic acid was mixed with 180 µL of oxalyl chloride with a catalytic amount of DMF in DCM for 3 hours. The reaction was concentrated under reduced pressure and dissolved in 3 mL of DCM. 270 mg of 2-amino-*p*-cresol and 300 µL of pyridine were added on ice. The reaction was allowed to warm to room temperature over 16 hours. The reaction was diluted in DCM and washed twice with 1 M HCl followed by a wash with brine. The reaction was dried with magnesium sulfate and concentrated and purified via column chromatography using 8:1 hexane ethyl acetate to give 60% yield.

^{1}H NMR (400 MHz, Chloroform-*d*) δ 8.41 (s, 1H), 7.26 (s, 1H), 7.23 – 7.11 (m, 3H), 6.90 (d, *J* = 2.5 Hz, 2H), 6.62 (s, 1H), 3.06 (t, *J* = 7.7 Hz, 2H), 2.69 (dd, *J* = 8.5, 6.8 Hz, 2H), 2.34 (s, 3H), 2.22 (s, 3H). M/Z=270.1495

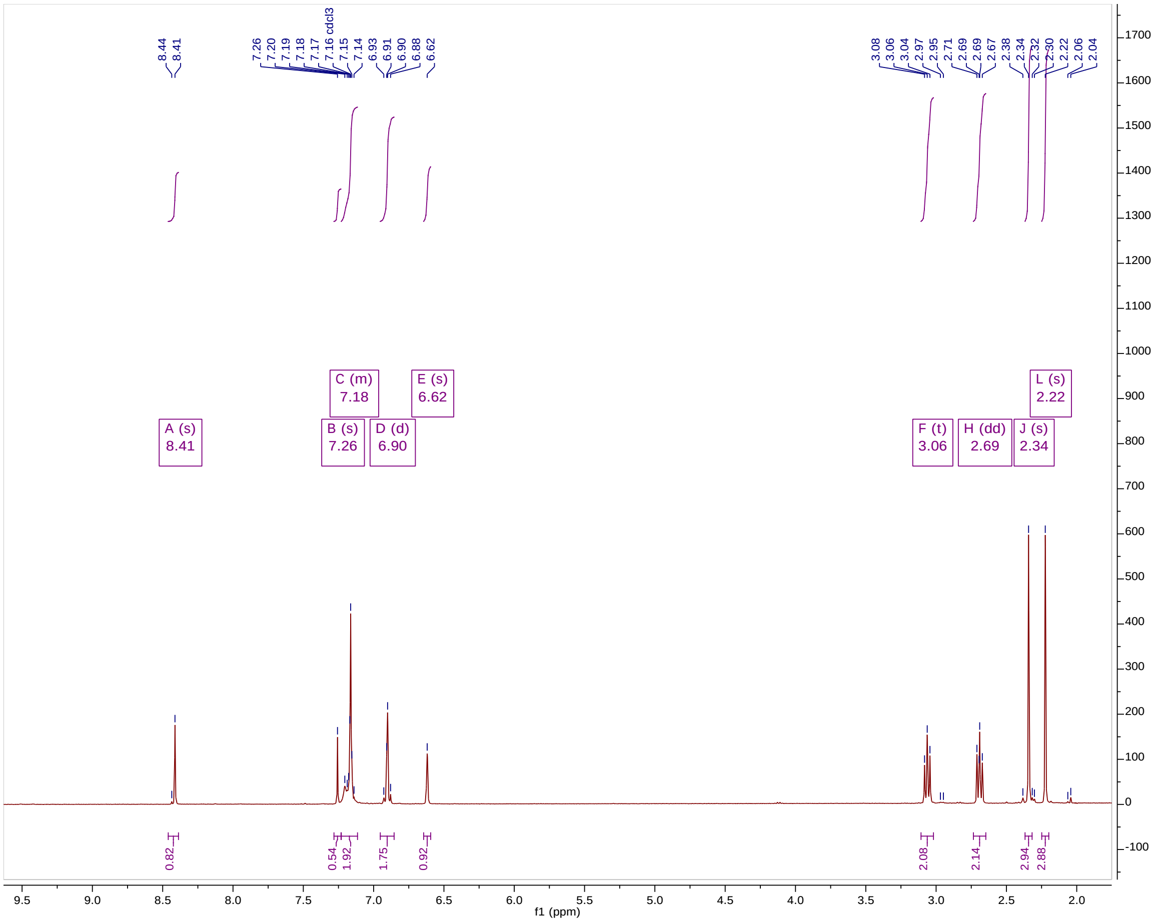

AA147-16

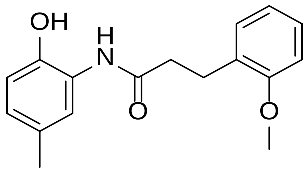

186 mg of 3-(2-methoxyphenyl)propionic acid was mixed with 180 µL of oxalyl chloride with a catalytic amount of DMF in DCM for 3 hours. The reaction was concentrated under reduced pressure and dissolved in 3 mL of DCM. 266 mg of 2-amino-*p*-cresol and 250 µL of pyridine were added on ice. The reaction was allowed to warm to room temperature over 16 hours. The reaction was diluted in DCM and washed twice with 1 M HCl followed by a wash with brine. The reaction was dried with magnesium sulfate and concentrated and purified via column chromatography using 8:1 hexane ethyl acetate to give 52% yield.

1H NMR (400 MHz, Chloroform-d) δ 8.25 (s, 1H), 7.61 (s, 1H), 7.25 – 7.12 (m, 2H), 7.03 (s, 1H), 6.90 (ddd, J = 24.9, 18.2, 9.3 Hz, 4H), 3.82 (s, 3H), 2.95 (t, J = 7.4 Hz, 2H), 2.68 (s, 2H), 2.26 (s, 3H). M/Z=286.1442

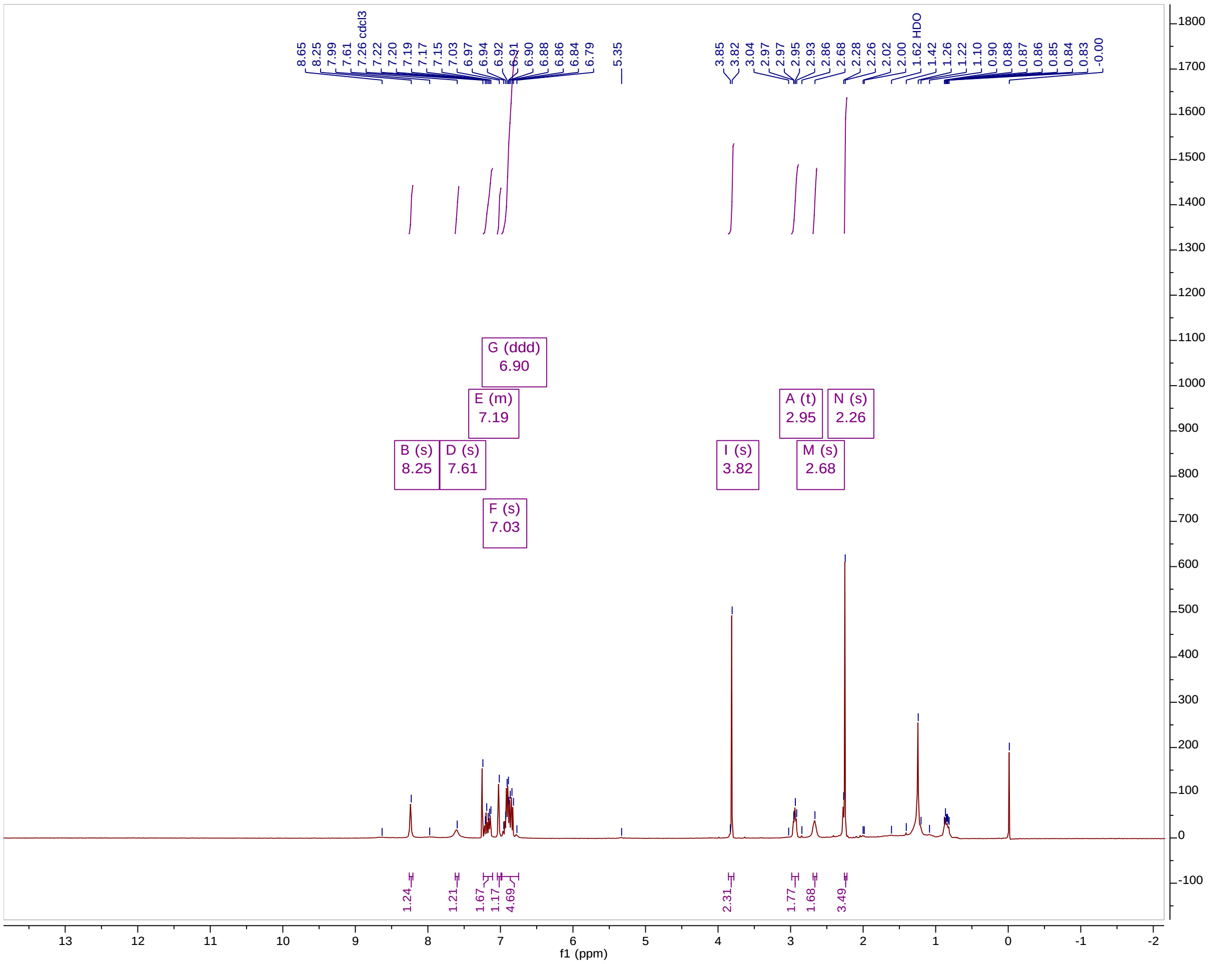

AA147-17

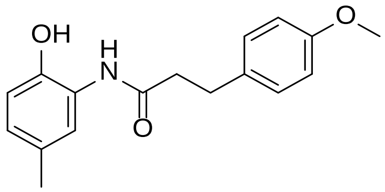

^{1}H NMR (400 MHz, Chloroform-*d*) δ 8.43 (s, 1H), 7.15 (t, *J* = 8.3 Hz, 3H), 6.95 – 6.75 (m, 4H), 6.57 (s, 1H), 3.79 (s, 3H), 3.02 (t, *J* = 7.3 Hz, 2H), 2.71 (t, *J* = 7.4 Hz, 2H), 2.22 (s, 3H).

186 mg of 3-(4-methoxypheny)propionic acid was mixed with 180 µL of oxalyl chloride with a catalytic amount of DMF in DCM for 3 hours. The reaction was concentrated under reduced pressure and dissolved in 3 mL of DCM. 275 mg of 2-amino-*p*-cresol and 140 µL of TEA were added on ice. The reaction was allowed to warm to room temperature over 16 hours. The reaction was diluted in DCM and washed twice with 1 M HCl followed by a wash with brine. The reaction was dried with magnesium sulfate and concentrated and purified via column chromatography using 4:1 hexane ethyl acetate to give 44% yield. M/Z=286.1424

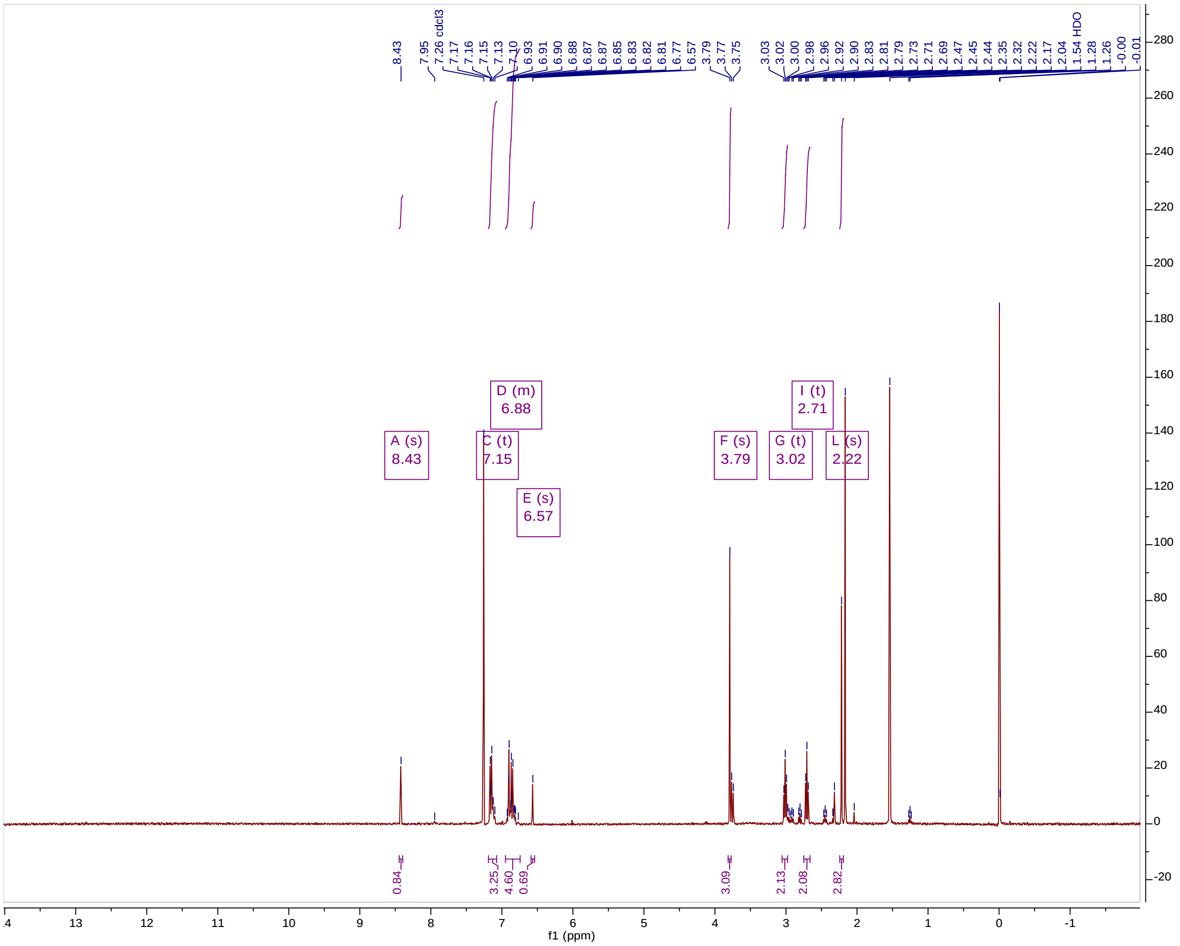

AA147-18

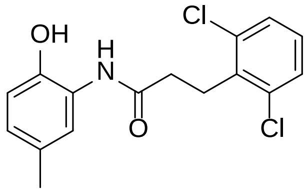

^{1}H NMR (400 MHz, Chloroform-*d*) δ 8.33 (s, 1H), 7.40 – 7.28 (m, 3H), 7.13 (t, *J* = 8.0 Hz, 1H), 6.92 (s, 2H), 6.80 (s, 1H), 3.43 – 3.34 (m, 2H), 2.75 – 2.66 (m, 2H), 2.25 (s, 3H).

220 mg of 3-(2, 6-dichlorophenyl)propionic acid was mixed with 180 µL of oxalyl chloride with a catalytic amount of DMF in DCM for 3 hours. The reaction was concentrated under reduced pressure and dissolved in 3 mL of DCM. 260 mg of 2-amino-*p*-cresol and 300 µL of pyridine were added on ice. The reaction was allowed to warm to room temperature over 16 hours. The reaction was diluted in DCM and washed twice with 1 M HCl followed by a wash with brine. The reaction was dried with magnesium sulfate and concentrated and purified via column chromatography using 4:1 hexane ethyl acetate to give 61 mg 47% yield. M/Z=324.0561

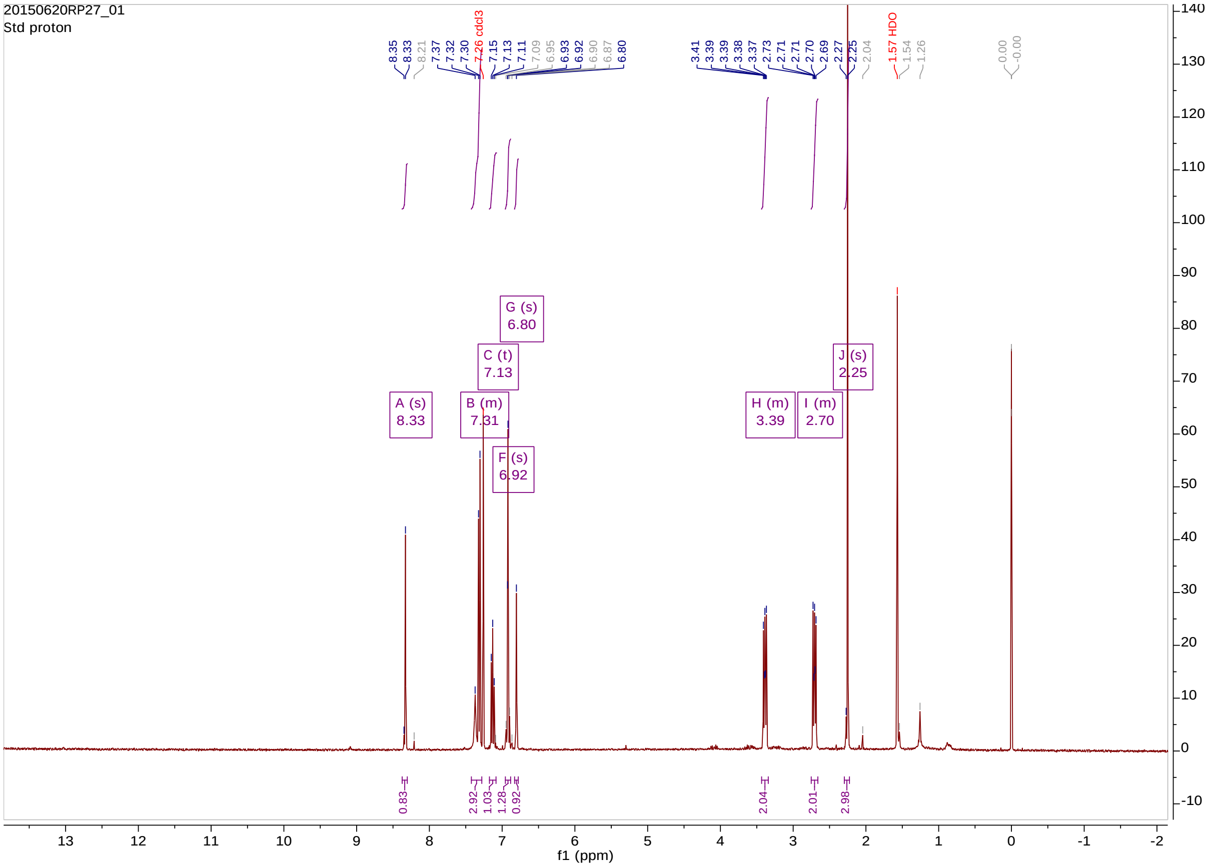

AA147-20

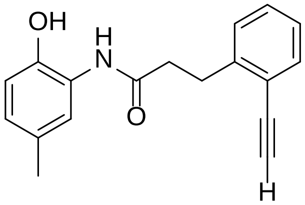

135 mg (0.4 mmol) of AA147-10 was mixed with 60 µL (0.8 mmol) of chloromethyl methyl ether and 210 µL (1.2 mmol) of disopropylethyl amine in DCM on ice warming to room temperature for 18 hours. The reaction was washed with saturated ammonium chloride followed by a wash with brine, dried with magnesium sulfate, concentrated and purified with 4:1 hexane ethyl acetate to give 85% yield of AA147-20a. M/Z=280.1335

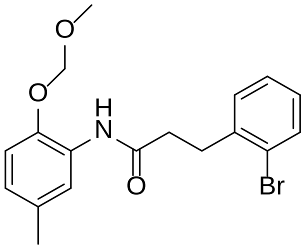

^{1}H NMR (400 MHz, Chloroform-*d*) δ 8.38 (s, 1H), 7.92 (s, 1H), 7.67 (d, *J* = 7.8 Hz, 1H), 7.45 (d, *J* = 7.2 Hz, 1H), 7.36 (t, *J* = 7.3 Hz, 1H), 7.20 (d, *J* = 14.7 Hz, 1H), 7.12 (d, *J* = 8.2 Hz, 1H), 6.94 (d, *J* = 7.9 Hz, 1H), 5.27 (s, 2H), 3.59 (s, 3H), 3.32 (t, *J* = 7.5 Hz, 2H), 2.86 (t, *J* = 7.5 Hz, 2H), 2.44 (s, 3H). M/Z=378.0

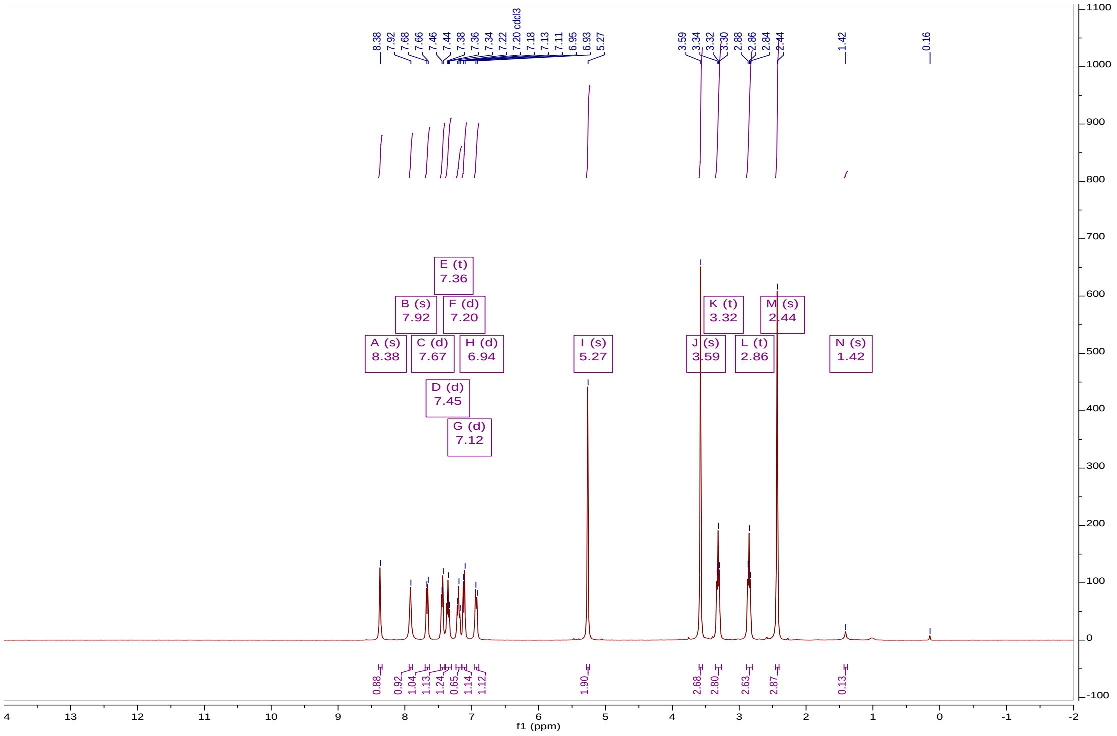

80 mg of AA147-20a was mixed with 11.5 mg (0.03 mol) of $Pd(PhCN)_2Cl_2$ and 3.8 mg (0.02 mol) of CuI. The flask was purged and backfilled with argon three times followed by the addition of 42 µL of 0.3 mol of diisopropylamine and 1 mL of anhydrous dioxane. 210 µL of 10% wt phosphine in hexane was added to the solution. 71 µL (0.5 mmol) of trimethyl silyl acetylene was dripped into the reaction on ice. The reaction proceeded to for 10 hours and was diluted into ethyl acetate. The reaction was filtered through a pad of silica gel, concentrated and purified with 25:1 toluene: ethyl acetate to give AA147-20b 85% yield.

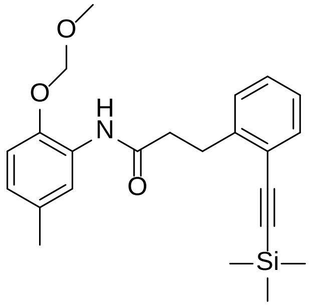

^{1}H NMR (400 MHz, Chloroform-*d*) δ 8.38 (s, 1H), 7.81 (s, 1H), 7.60 (d, *J* = 7.6 Hz, 1H), 7.44 – 7.32 (m, 2H), 7.36 – 7.25 (m, 1H), 7.13 (d, *J* = 8.3 Hz, 1H), 6.94 (dd, *J* = 8.4, 2.0 Hz, 1H), 5.25 (s, 2H), 3.57 (s, 3H), 3.37 (t, *J* = 7.7 Hz, 2H), 2.91 (t, *J* = 7.7 Hz, 2H), 2.45 (s, 3H) 0.41 (s, 9H). M/Z=396.2

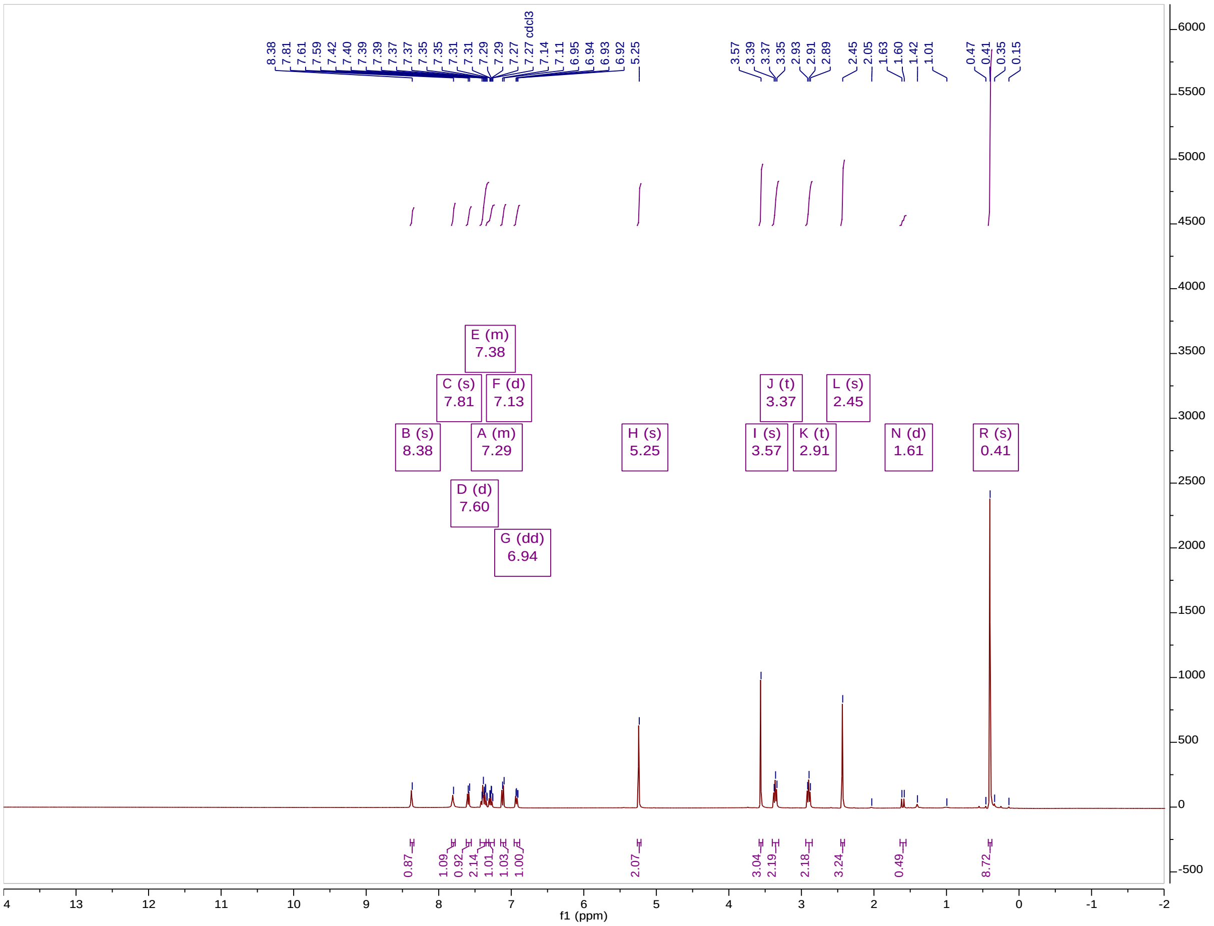

The ether protecting group of AA147-20b was removed by the addition of 5 equivalence of HCl in MeOH mixing for 4 hours. The reaction was washed with 1 M HCl followed by a wash with brine, dried with magnesium sulfate, concentrated, and purified with 9:1 Hexane: Ethyl acetate to give AA147-20c with a 78% yield.

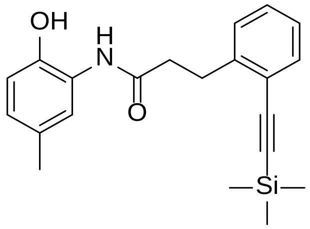

^{1}H NMR (400 MHz, Chloroform-*d*) δ 8.48 (s, 1H), 7.48 (d, *J* = 7.4 Hz, 1H), 7.32 – 7.14 (m, 3H), 6.88 (s, 2H), 6.62 (s, 1H), 3.21 (t, *J* = 7.5 Hz, 2H), 2.79 (t, *J* = 7.5 Hz, 2H), 2.21 (s, 3H), 1.26 (s, 0H), 0.26 (s, 9H). M/Z=352.2

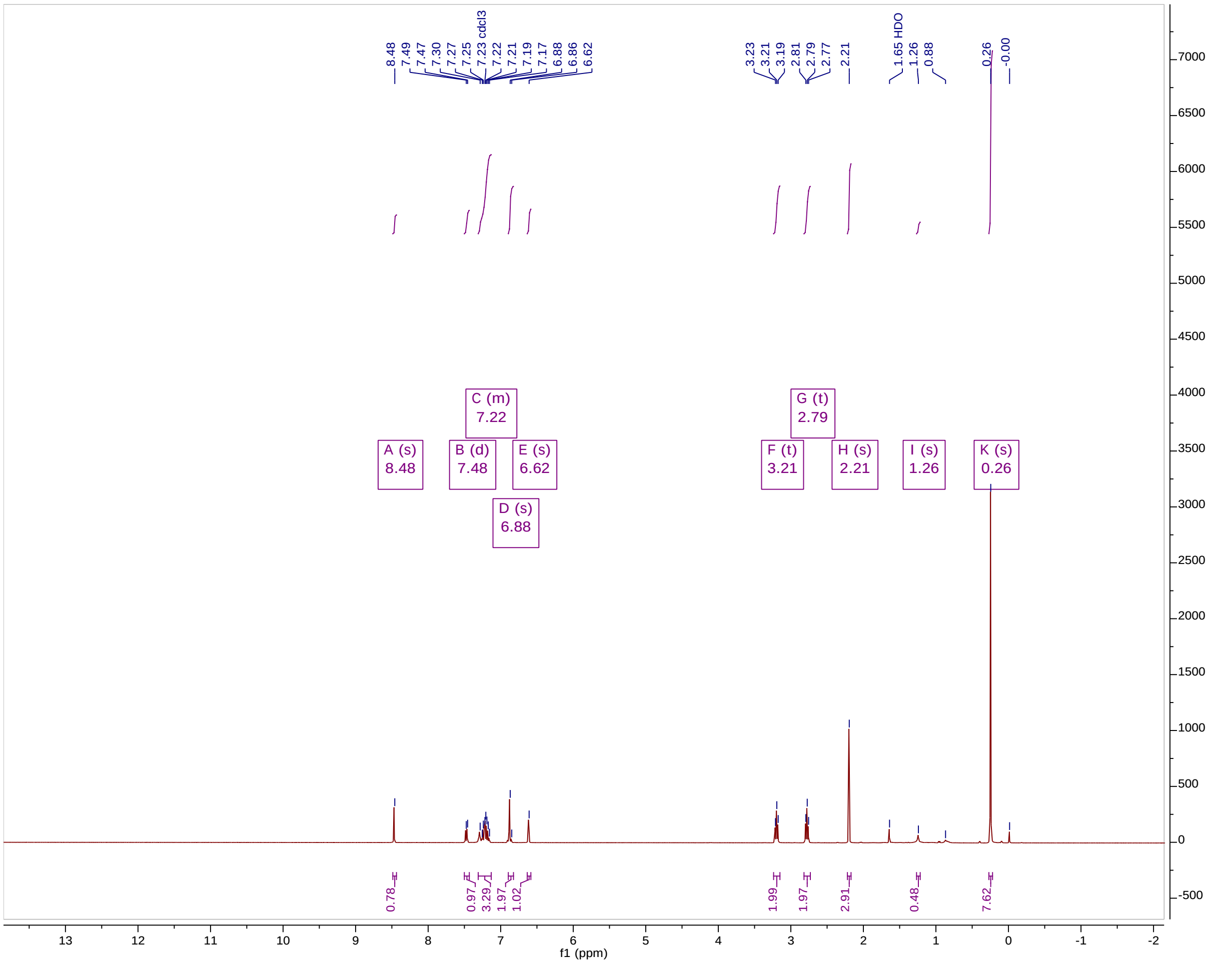

AA147-20c was mixed with 5 equivalence of potassium carbonate in methanol for 2 hours. The reaction was quenched with 1 M HCl and was washed twice with brine. The reaction was dried with magnesium sulfate, concentrated and purified with 6:1 hexane: ethyl acetate to give 84% yield.

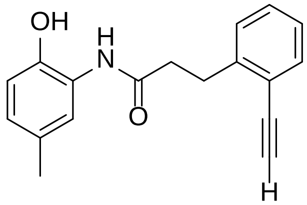

^{1}H NMR (400 MHz, Chloroform-*d*) δ 8.40 (s, 1H), 7.52 (d, *J* = 7.5 Hz, 1H), 7.34 – 7.17 (m, 4H), 6.90 (d, *J* = 2.6 Hz, 2H), 6.65 (s, 1H), 3.34 (s, 1H), 3.24 (t, *J* = 7.6 Hz, 2H), 2.80 (t, *J* = 7.6 Hz, 2H), 2.22 (s, 3H). M/Z=280.1

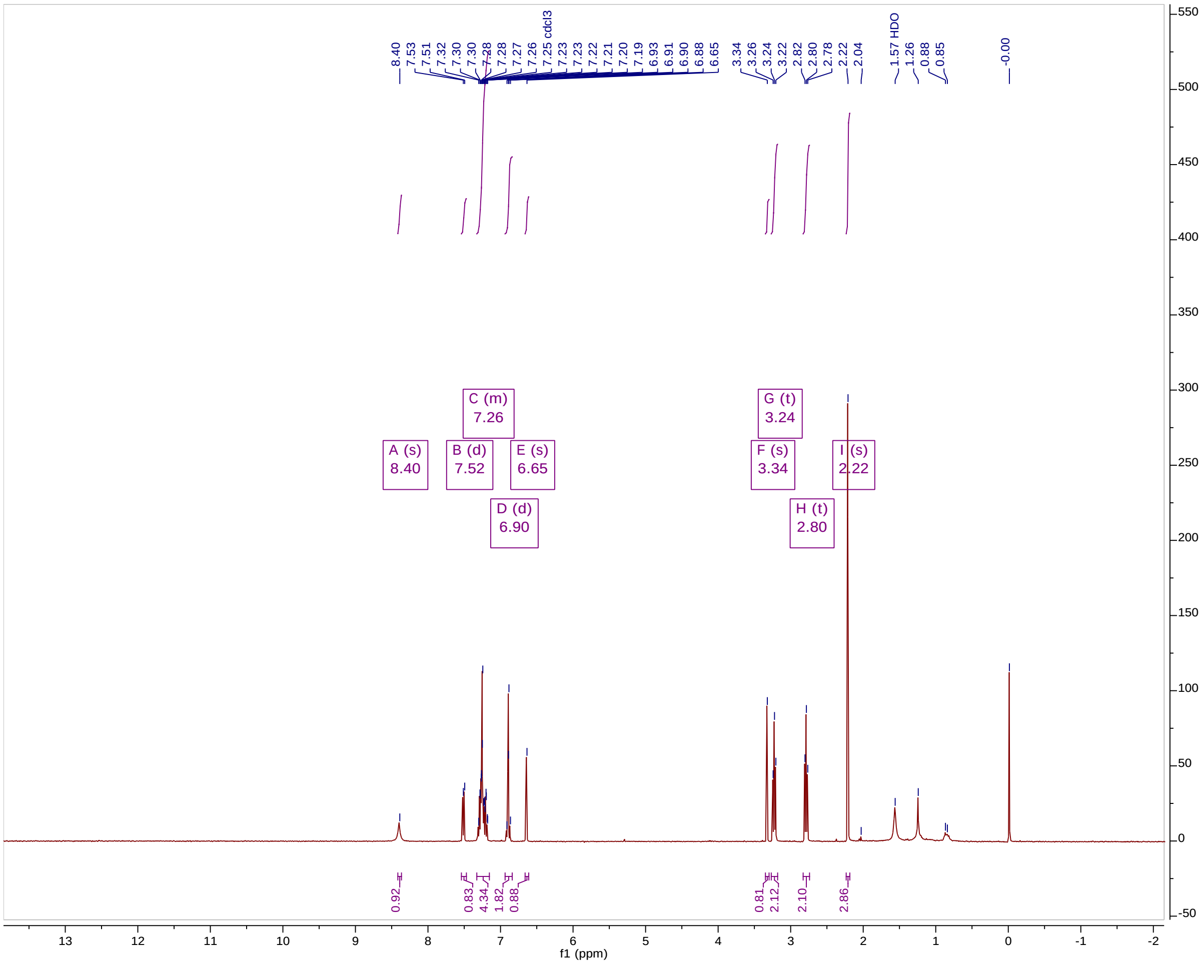

Supplement: Supplementary file 1. [file elife-37168-supp1.pdf]
